# Investigation of Ensemble Machine Learning Models for Estimating the Ultimate Strain of FRP-Confined Concrete Columns

**DOI:** 10.3390/ma19010189

**Published:** 2026-01-04

**Authors:** Quang Trung Nguyen, Anh Duc Pham, Quynh Chau Truong, Cong Luyen Nguyen, Ngoc Son Truong, Anh Duc Mai

**Affiliations:** 1 Construction Management Division, The University of Da Nang—University of Science and Technology, Da Nang 550000, Vietnam; nqtrung@dut.udn.vn (Q.T.N.); paduc@dut.udn.vn (A.D.P.); tqchau@dut.udn.vn (Q.C.T.); tnson@dut.udn.vn (N.S.T.); 2 Construction Informatics Division, The University of Da Nang—University of Science and Technology, Da Nang 550000, Vietnam; ncluyen@dut.udn.vn

**Keywords:** FRP fiber, FRP-confined concrete, ultimate strain of FRP-confined concrete, estimating the strain capacity of FRP-confined concrete, machine learning model, artificial intelligence

## Abstract

Accurately predicting the ultimate strain of fiber-reinforced polymer (FRP)-confined concrete columns is essential for the widespread application of FRP in strengthening reinforced concrete (RC) columns. This study comprehensively investigates the performance of ensemble machine learning (ML) models in estimating the ultimate strain of FRP-confined concrete (FRP-CC) columns. A dataset of 547 test results of the ultimate strain of FRP-CC columns was collected from the literature for training and testing ML models. The four best single ML models were used to develop ensemble models employing voting, stacking and bagging techniques. The performance of the ensemble models was compared with 10 single ML and 11 empirical strain models. The study results revealed that the single ML models yielded good agreement between the estimated ultimate strain and the test results, with the best single ML models being the K-Star, k-Nearest Neighbor (k-NN) and Decision Table (DT) models. The three best ensemble models, a stacking-based ensemble model comprising K-Star, k-NN and DT models; a stacking-based ensemble model comprising K-Star and k-NN models and a voting-based ensemble model comprising K-Star and k-NN models, achieved higher estimation accuracy than the best single ML model in estimating the strain capacity of FRP-CC columns.

## 1. Introduction

Fiber-reinforced polymer (FRP) materials have been widely applied in the construction industry over the last 30 years owing to their advantages of high tensile capacity, low weight, quick and simple installation and minimal maintenance duration [1,2,3,4]. One important application of FRP materials is as a retrofitting material for existing reinforced concrete (RC) columns to improve their load-bearing capacity and deformation. Extensive research investigations have focused on the performance of FRP-confined concrete columns (hereafter referred to as FRP-CC columns for brevity) in an attempt to gain insight into their behavior and provide an accurate estimation of the ultimate capacity (compressive strength/strength capacity and ultimate strain/strain capacity) of FRP-CC columns [5,6,7,8]. Thanks to these comprehensive research investigations, the response of FRP-CC columns is now well established, and over 90 stress–strain models have been proposed [9,10,11,12].

A few research studies have examined the precision of available stress–strain models in estimating the ultimate capacity of FRP-CC columns [9,13,14]. These investigations have indicated that, while the available FRP-CC stress–strain models have yielded satisfactory estimation of the strength capacity of FRP-CC, their prediction of the strain capacity of FRP-CC is far less reliable [9,13,14]. Bisby et al. [13] assessed the estimation accuracy of the available stress–strain models using the test results of 200 FRP-CC columns compiled from 20 experimental studies. It was found by Bisby et al. [13] that the average absolute error (AAE) for the best strength model was approximately 14%, while the AAE for the best strain model exceeded 35%. Similarly, Ozbakkaloglu et al. [9] compared the performance of the FRP-CC stress–strain models using an extensive database assembled from the literature. Ozbakkaloglu et al. [9] found that the AAE of the selected empirical strength models performed over 705 test data points of FRP-CC columns was 18.6%, while the AAE of the selected empirical strain models constructed over 527 test data points was 53.0%. Evaluating the estimation accuracy of empirical strength and strain models was also carried out by Ozbakkaloglu and Lim [14]. Ozbakkaloglu and Lim [14] reported that the best strength model attained an AAE of about 12% while the best strain model received an AAE of more than 26%.

A large discrepancy between the estimated values obtained by the empirical strain models and test results can be attributed to the following reasons: (1) The majority of the best empirical strain expressions $\left(\varepsilon_{cu}\right)$ is in non-linear forms and associated with the confinement ratio ($f_{l}/f_{co})$ [14]. The maximum lateral confining pressure ($f_{l})$ provided by the FRP jacket is largely influenced by the FRP hoop rupture strain $\left(\varepsilon_{fe}\right)$, which is calculated as the product of the FRP jacket’s ultimate tensile strain $\left(\varepsilon_{fu}\right)$ and the strain reduction factor $\left(k_{\varepsilon }\right)$ [9]. The $k_{\varepsilon }$ was assumed to be a constant value in most of the existing strain models, while this factor was found to vary in the previous studies [14,15,16,17,18]. Thus, existing empirical strain models are incapable of accurately estimating the strain capacity of FRP-CC columns. (2) Since empirical strain models are usually directly derived and calibrated from test results, their accuracy is strongly tied to the test database utilized for the model’s formulation. Consequently, the empirical strain models are capable of accurately estimating the strain capacity of FRP-CC columns when evaluated against the same dataset used for model development; however, they yield unsatisfactory estimation of the strain capacity of FRP-CC columns when applied to a broader and more diverse test database. (3) The ultimate strain of FRP-CC columns has been found to be influenced by different parameters, such as the compressive strength ${(f}_{co})$ and ultimate strain $\left(\varepsilon_{co}\right)$ of control concrete [14,19], FRP hoop rupture strain $\left(\varepsilon_{fe}\right)$, the maximum lateral confining pressure ${(f}_{l})$, the properties of the FRP jacket and unknown parameters [20]. However, the empirical strain models are incapable of reflecting the influence of all the parameters and their influence levels. This contributes to the uncertainty of empirical strain models in estimating the strain capacity of FRP-CC columns. Due to the limitations of empirical models in estimating the strain capacity of FRP-CC columns, new approaches for estimating the strain capacity of FRP-CC columns are needed.

Machine learning (ML) models have recently seen extensive application in addressing a wide range of challenging civil engineering tasks, including material [21,22,23,24,25,26], geotechnical [27,28] and structural engineering [29,30,31,32], as well as construction management [33,34]. This rapid adoption is driven by their advantages over traditional statistical approaches, possessing ability to learn sophisticated and non-linear relationships directly from data without the need for pre-defined mathematical equations [35]. The ML models have been proven to be highly effective tools for forecasting the strength capacity of FRP-CC columns [36,37,38,39,40,41]. Several research studies, including Cevik and Guzelbey [37], Jalal and Ramezanianpour [38], Naderpour et al. [39] and Elsanadedy et al. [40], estimated the strength capacity of FRP-CC columns using artificial neural network (ANN) models and then compared their performance with that of empirical strength models. The ANN model estimated the strength capacity of FRP-CC columns well and outperformed existing empirical strength models. Cevik et al. [42] employed the genetic programming and stepwise regression algorithms to develop empirical expressions for estimating the strength capacity of FRP-CC columns. It is reported in Cevik et al. [42] that the developed empirical expression performed better than existing empirical strength models. The application of soft computing models (e.g., ANN, stepwise regression, neuro-fuzzy and genetic programming models) to estimate the strength capacity of FRP-CC columns was also undertaken by Cevik [43]. It was found in Cevik [43] that soft computing models provided better estimation accuracy than empirical strength models, with the ANN model achieving the best performance.

Mozumder et al. [44] applied the ANN and support vector regression (SVR) models in estimating the strength capacity of FRP-CC columns. Mozumder et al. [44] found that the single ML models provided a good estimation of the strength capacity of the FRP-CC columns and obtained higher estimation accuracy than the existing empirical strength models, with the best ML model obtained by the SVR model. Yu and Hu [45] estimated the strength capacity of CFRP-CC columns using five single ML models: linear regression (LR), Ridge Regression (RR), decision tree (DT), Random Forest (RF) and ANNs. The study results of Yu and Hu [45] showed that the best performance model among the single ML models was the ANN. Tao et al. [41] compared the performance of Extreme gradient boosting (EGBoost) with three ML models of Multivariable Adaptive Regression Spline (MARS), Extreme Learning Machine (EML), Random Forest GenRator (Ranger) and found that the EGBoost model performed better than the three ML models and provided a good agreement between the estimated and observed strength capacity of FRP-CC columns. Zeng et al. [46] adopted the Conditional Tabular Generative Adversarial Network (CTGAN) to generate synthetic data for training and testing the single ML models of RF, Gradient Boosting decision tree (GBDTR), EGBoost and ANN in estimating the strength capacity of FRP-CC columns. These single ML models obtained high estimation accuracy for the strength capacity of FRP-CC columns and also surpassed the existing empirical strength models. Khodadadi et al. [47] developed a hybrid ML model by integrating the Particle Swarm Optimization into the Categorical Boosting algorithm (PSO-CatBoost) for predicting the strength capacity of FRP-CC columns. It has been found by Khodadadi et al. [47] that the developed hybrid model outperformed the six single ML models of CatBoost, SgBoost, AdaBoost, GBoost, Extra Trees and Random Forest as well as six empirical strength models in estimating the strength capacity of FRP-CC columns. Hu et al. [48] adopted six ML models consisting of linear regression, Ridge Regression, ANN, Decision Tree, Random Forest and eXtreme Gradient Boosting for estimating the strength capacity of FRP-CC columns. It was revealed in Hu et al. [48] that the eXtreme Gradient Boosting performed the best in estimating the strength capacity of FRP-CC columns and provided a good estimation of the strength capacity of FRP-CC columns.

The application of ML models to estimate the strain capacity of FRP-CC columns has been reported in a few research studies [49,50,51]. Keshtegar et al. [49] developed five types of strain and strength models for ultimate conditions of FRP-CC columns based on the dynamic harmony search (DHS) algorithm. A dataset of 780 test results of FRP-CC columns was collected from the literature to validate the estimation accuracy of the existing developed empirical models. Keshtegar et al. [49] revealed that the proposed empirical models estimated the ultimate conditions of FRP-CC columns well and surpassed existing empirical models in estimating the ultimate conditions of FRP-CC columns. Lim et al. [50] applied genetic programming to develop empirical strength and strain models for estimating the strength and ultimate strain, respectively, of FRP-CC columns. A dataset of 753 compressive strength and 511 ultimate tensile strain of FRP-CC columns was compiled in Lim et al. [50] for validating the developed empirical models and available empirical models. It was reported that the proposed empirical equation performed slightly lower than the best empirical models in estimating both the strength and strain capacity of FRP-CC columns. Mansouri et al. [51] applied four ML models, including neuro fuzzy, neural network, multivariable adaptive regression splines (MARS) and M5 models, in estimating the ultimate condition (strength and ultimate strain) of FRP-CC columns. A dataset of 1079 test results of FRP-CC columns was also assembled in Mansouri et al. [51] to examine the estimation accuracy of the ML models and empirical strain models. It can be seen in Mansouri et al. [51] that, for estimating both the strength and strain capacity of FRP-CC columns, the ANN model performed the best and its estimation accuracy was higher than that of the best empirical model, while the performance of the three remaining ML models in predicting the ultimate conditions of FRP-CC columns were not as good as the best empirical model.

As presented in the literature review, the majority of available investigations of ML models on FRP-CC columns have been applied to predicting the strength capacity of FRP-CC columns, while a minimal number of studies of ML models have been employed for estimating the strain capacity of FRP-CC columns. Thus, further investigations into the applications of ML models for estimating the strain capacity of FRP-CC columns are needed to ensure their reliability. It is also observed that a comprehensive evaluation of the prediction performance of various ML models in estimating the strain capacity of FRP-CC columns would provide an overall review for design engineers in selecting the most suitable ML models, which has not been available in the literature. Furthermore, the investigation of the performance of ensemble ML models in estimating the strain capacity of FRP-CC columns has not yet been conducted. It is noted that ensemble ML models have been proven to perform better than single ML models in estimating the compressive strength of high-performance concrete [32] and energy consumption in buildings [31]. Thus, this study investigates the estimation performance of ensemble ML models in estimating the strain capacity of FRP-CC columns. The performance of ensemble ML models was compared with that of various single ML and existing empirical strain models.

The ensemble models were developed using the following procedure: a database comprising 547 data points on the ultimate strain of FRP-CC columns was first assembled from available studies in the literature. Eleven empirical and ten single ML models were used to predict the strain capacity of FRP-CC columns. The performance of ML models was compared with that of the empirical models using five statistical indicators: correlation coefficient (R), mean absolute error (MAE), root mean square error (RMSE), mean absolute percentage error (MAPE) and synthetic indicator (SI). The four best ML models, which performed better than or similarly to the best empirical model, were selected to develop ensemble models using voting, stacking and bagging techniques. The prediction accuracy of the ensemble models was compared to that of the best single ML models to determine the best ensemble models.

## 2. Prediction Models

### 2.1. Empirical Models for Predicting the Ultimate Strain of FRP-Confined Concrete

#### 2.1.1. Mechanism Confinement of FRP-Confined Concrete

**Figure 1:**
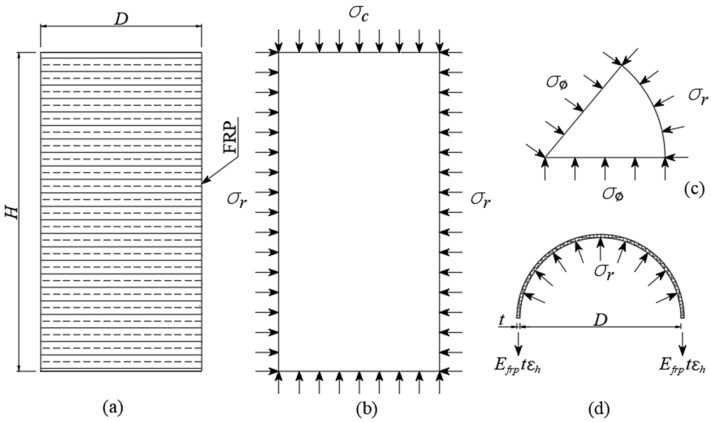
Confining mechanism of FRP-CC column: (**a**) FRP-confined concrete column; (**b**) confining action of concrete core; (**c**) confining action of concrete segment; (**d**) confining action of FRP jacket.

For an FRP-confined concrete column under axial compression, the lateral confining pressure ($\sigma_{r})$ exerted by the FRP jacket, which is assumed to distribute uniformly around the circumference of the concrete core, inhibits the transverse dilation of concrete, as schematically described in Figure 1. The $\sigma_{r}$ is passive, as it arises as a result of the transverse dilation of the concrete core and can be determined based on the force equilibrium, as presented in Equation (1).

(1) $$\sigma_{r}=\frac{2\sigma_{h}t_{f}}{D}=\frac{2E_{f}{nt}_{f}\varepsilon_{f}}{D}$$

where $\sigma_{h}$ denotes the tensile stress in the FRP jacket in the hoop direction; D denotes the diameter of the confined concrete specimens; $nt_{f}$, $E_{f}$ and $\varepsilon_{f}$ denote the total thicknesses, elastic modulus and tensile strain of the FRP jacket and n denotes the number of FRP plies in the FRP jacket.

Due to the linear elastic property of the FRP material, the $\sigma_{h}$ is proportional to the hoop strain $\left(\varepsilon_{f}\right)$ and obtains the maximum value $\left(f_{l}\right)$ at the rupture of the FRP jacket. Accordingly, the $f_{l}$ is determined based on the rupture strain of the FRP jacket $\left(\varepsilon_{fe}\right)$, as given by the following:

(2) $$f_{l}=\;\frac{2E_{f}nt_{f}}{D}\varepsilon_{fe}$$

#### 2.1.2. Empirical Models for Ultimate Strain of FRP-Confined Concrete

It has been reported that the rupture strain of the FRP jacket $\left(\varepsilon_{fe}\right)$ is smaller than the rupture strain of FRP determined by the coupon test $\left(\varepsilon_{fu}\right)$. The discrepancy between $\varepsilon_{fe}$ and $\varepsilon_{fu}$ was measured by the strain reduction factor $\left(k_{\varepsilon }\right)$, which was determined by dividing $\varepsilon_{fe}$ by $\varepsilon_{fu}$. Based on the test results of an experimental program, Harries and Carey [17] concluded that $k_{\varepsilon }$ varied from 0.7 to 0.9, which was influenced by the thickness of the FRP jacket. Lam and Teng [15] suggested taking a constant value of 0.63 for the $k_{\varepsilon }$ after examining the $\varepsilon_{fe}$ of the available studies. Ozbakkaloglu and Lim [14] found that the $k_{\varepsilon }$ was affected by the compressive strength of control concrete $\left(f_{co}\right)$ and the $E_{f}$. Ozbakkaloglu and Lim [14] suggested determining the $k_{\varepsilon }$ using the following equation.

(3) $$k_{\varepsilon }=0.9-2.3f_{co}10^{-3}-0.75E_{f}10^{-6}$$

Wu and Jiang [52] carried out an experimental program and suggested that the $k_{\varepsilon }$ of about 0.9 should be used for determining the $f_{l}$. Based on an experimental study, Smith et al. [18] found that the $k_{\varepsilon }$ obtained a value of 0.81. As the $k_{\varepsilon }$ was different in the literature, some available empirical strain models have been developed based on the $\varepsilon_{fe}$, while others have been developed based on the $\varepsilon_{fu}$. Table 1 lists 11 selected empirical models for the ultimate strain of FRP-confined concrete. The prediction performance of these empirical strain models was compared to that of the machine learning models. Three out of eleven empirical strain models were adopted in three design codes for concrete structures externally strengthened by FRP, and eight remaining empirical models were shown to provide satisfactory accuracy in predicting the ultimate strain of FRP-confined concrete columns.

### 2.2. Machine Learning Models

#### 2.2.1. Linear Regression (LR)

The LR model is a fundamental supervised ML algorithm, utilized to estimate a target label, which is a continuous numeric value, based on a linear relationship with one or more input variables. For multiple input variables ($x_{1},x_{2},\ldots ,x_{n})$, the relationship between the predictors and output is presented by the following linear expression.

(4) $$y^{\prime }=\beta_{0}+\beta_{1}x_{1}+\beta_{2}x_{2}+\cdots +\beta_{n}x_{n}$$

where $y^{\prime }$ and n, respectively, denote the predicted label and number of data points.

The goal of the training process in the ML model is to find the optimal values for the bias $\left(\beta_{0}\right)$ and weight of input variables $(\beta_{1},\beta_{2}$,…, $\beta_{n})$. The above coefficient can be found by minimizing the sum of squared errors (SSE) between the model’s prediction and true observation, also known as the cost function:

(5) $$SSE=\sum_{i=1}^{n}{\left(y_{i}-y^{\prime }\right)}^{2}$$

#### 2.2.2. Gaussian Process (GP)

A GP model is a Bayesian non-parametric ML model primarily used for regression. Unlike the linear regression, which provides a best-fit line, a Gaussian Process treats estimation as a distribution over possible functions, which gives a range of possible functions that could fit the data along with a probability for each. Accordingly, a Gaussian Process is considered a powerful and flexible ML model. The Gaussian Process is fully specified by a mean function $\left[m\left(x\right)\right]$ and a covariance function (kernel) $\left[k\left(x,x^{\prime }\right)\right]$, as follows:

(6) $$f(x)\sim \;GP(m\left(x\right),k(x,\;x^{\prime }))$$

The expected value of the underlying $f\left(x\right)$ at point x is described by the mean function $m\left(x\right)$, written as follows:

(7) $$m\left(x\right)=E[f(x)]$$

For simplicity and flexibility, the mean function is often assumed to be zero $m\left(x\right)$ = 0. The kernel $k\left(x,x^{\prime }\right)$ specifies how similar the function values are at two different inputs, $x\;and\;x^{\prime }$, controlling smoothness, shape and behavior of the predicted function, expressed as follows:

(8) $$k(x,\;x^{\prime })=E(\left[f\left(x\right)-m(x)\right])[f(x^{\prime })-m(x^{\prime })]$$

For a set of training data with input variables of $X=\left\{x_{1},x_{2},\ldots ,x_{n}\right\}$ and output variable of y, the Gaussian Process expresses the relationship between the predictors and output as follows:

(9) $$f={\left[f\left(x_{1}\right),f\left(x_{2}\right),\ldots ,f\left(x_{n}\right)\right]}^{T}\sim N(m,K)$$

(10) $$m={\left[m\left(x_{1}\right),m\left(x_{2}\right),\ldots ,m\left(x_{n}\right)\right]}^{T}$$

(11) $$K_{i.j}=k\left(x_{i},\;x_{j}\right)$$

#### 2.2.3. Artificial Neural Networks (ANNs)

**Figure 2:**
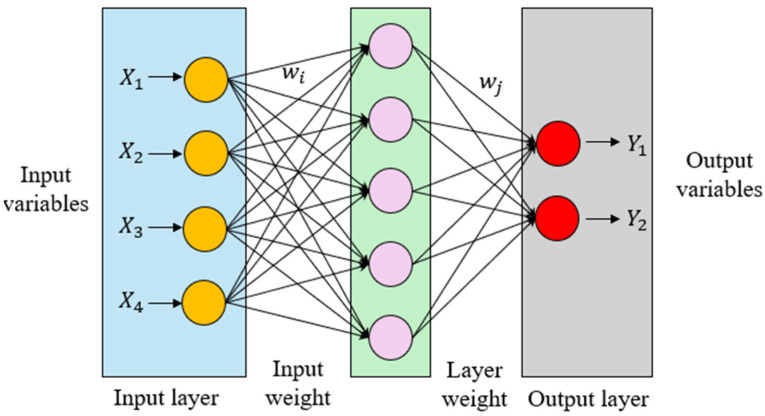
Architecture of conventional artificial neural network model.

The ANNs are algorithms designed to mimic the functioning of the brain’s neural architecture. ANN models comprise units considered as artificial neurons, which are interconnected to each other, working together to recognize patterns and learn complex relationships from data. The processing units are organized into a number of layers, which are classified into the input layer, hidden layer and output layer, forming the structure of a typical ANN model. A typical ANN model starts with an input layer, ends with an output layer and contains one or more hidden layers in the middle. Each artificial neuron of a layer receives multiple inputs, processes them and produces a single output to the next layer. The strength of the connections between neurons is represented by weights ($w_{i})$, considered the key parameters, which are adjusted during the learning process. The architecture of a conventional ANN employed to predict the ultimate strain of FRP-CC columns is graphically described in Figure 2.

In an ANN, each node processes its inputs by calculating a weighted combination, incorporating a bias and applying a non-linear activation function to pass the results to the neurons of the next layer. The computation performed at a neuron in the layer l is expressed in Equation (12) and the non-linear activation function $\sigma \left(.\right)$ is given in Equation (13):

(12) $$z_{i}^{\left(l\right)}=\sum_{j=1}^{n}w_{ij}^{\left(l\right)}a_{j}^{\left(l-1\right)}+b_{i}^{\left(l\right)}$$

(13) $$a_{i}^{\left(l\right)}=\sigma \left(z_{i}^{\left(l\right)}\right)$$

where $w_{ij}^{\left(l\right)}$ represents the learnable weight linking the neuron j in the preceding layer to the neuron i in the subsequent layer; $a_{j}^{\left(l-1\right)}$ denotes the activations from the previous layers; $b_{i}^{\left(l\right)}$, the bias term of the neuron i and $z_{i}^{\left(l\right)}$ denotes the input to the neuron i in layer l.

In the training process, a loss function is applied to assess the discrepancy between the model’s predictions $\left(y^{\prime }\right)$ and the observed values (y). The most common loss function is the Mean Squared Error (MSE).

(14) $$J(W,\;b)=\frac{1}{N}\sum_{i=1}^{N}{\left(y_{i}-y_{i}^{\prime }\right)}^{2}$$

The optimal ANN architecture in this study was found to be 7-15-1. The model was trained using the backpropagation algorithm with a sigmoid activation function. A similar ANN structure was adopted in Cevik and Guzelbey [37].

#### 2.2.4. Support Vector Regression (SVR)

**Figure 3:**
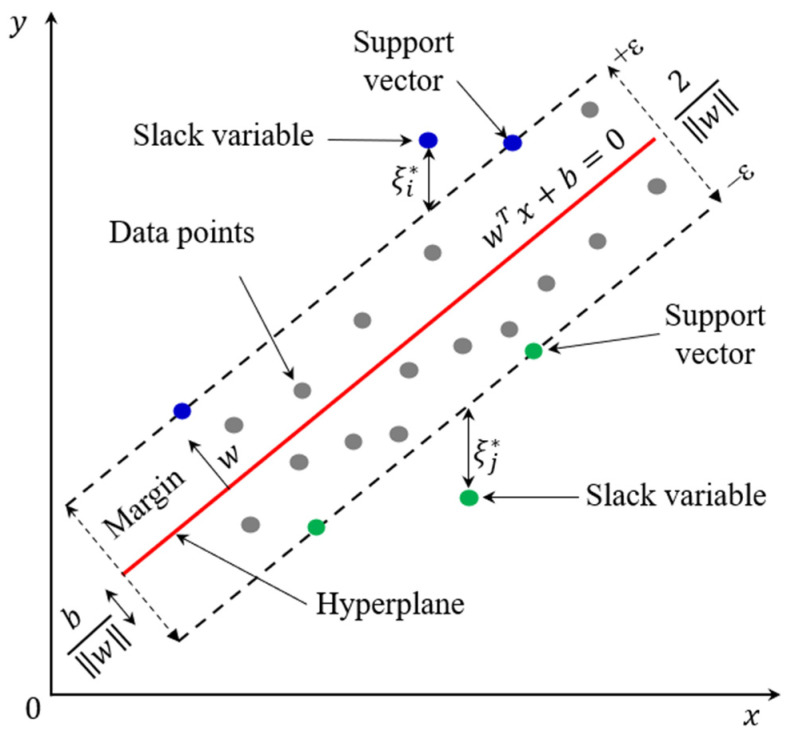
Schematic diagram of Support Vector Regression model.

The SVR is a variant of the Support Vector Machine, first introduced by Vapnik [62], adapted for regression tasks rather than classification. Unlike traditional regression models, such as the linear regression model, that attempt to minimize prediction errors directly, the SVR model aims to find an optimal regression function (hyperplane in high dimensions) that approximates the data within a certain allowable tolerance (ϵ). The model is designed to pay attention to data points that lie outside a specific margin, referred to as the ϵ-insensitive tube (Figure 3). This means that any prediction that falls within the margin ±ϵ of the actual value is considered error-free and incurs no penalty, while points that fall outside the ϵ-insensitive tube affect the loss function and are subjected to a penalty. The data points lying on the ϵ-insensitive tube define the position and orientation of the optimal hyperplane. This ϵ-insensitive approach makes SVR particularly effective for dealing with noisy data and complex as well as non-linear relationships. The prediction function $f\left(x\right)$ found by SVR can be expressed in a linear form, as shown in Equation (15).

(15) $$f\left(x\right)=w^{T}x+b$$

where $w^{T}$ and b, respectively, denote the weight vector and a bias term. The objective of the SVR model is to make the prediction function as flat as possible, which is equivalent to minimizing the norm of the weight vector, ${\left\|w\right\|}^{2}$. At the same time, the SVR model ensures that the deviation of the training data from the actual value must not exceed ϵ, while minimizing the ϵ-insensitive loss for points lying outside the ϵ-insensitive tube. To allow some errors outside the ϵ-insensitive tube, slack variables of $\xi_{i}\;{and\;\xi }_{i}^{*}$ are introduced for errors above the tube and errors below the tube, respectively. For the goal of balancing the model flatness and train errors, the optimization problem for a linear SVR model is formulated as follows:

(16) $${min}_{\omega ,b,\zeta ,\zeta^{*}}\;\frac{1}{2}{\left\|w\right\|}^{2}+C\sum_{i=1}^{n}\left(\zeta_{i}+\zeta_{i}^{*}\right)$$

subjected to the constraints:

(17) $$\left\{\begin{array}{c} y_{i}-\left(w^{T}x+b\right)\leq \epsilon +\zeta_{i}^{*}\\ \left(w^{T}x+b\right)-y_{i}\leq \epsilon +\zeta_{i}^{*}\\ \zeta_{i},\zeta_{i}^{*}\geq 0\;for\;i=1,2,\ldots ,n \end{array}\right.$$

where ${\left\|w\right\|}^{2}$ is used to measure the flatness of the function; C denotes the regularization coefficient that manages the balance between model smoothness and the degree of tolerance for deviations above ϵ; and n denotes the total training data points.

The model with the RBF kernel function, regularization parameter (C) of 3 and ϵ-insensitive loss of 0.001 was found to yield the best prediction of the strain capacity of FRP-CC columns.

#### 2.2.5. k-Nearest Neighbors (k-NN)

The k-NN algorithm is a straightforward and non-parametric ML model employed for both regression and classification tasks. Unlike many other ML models, which construct a generalizing model from the training dataset, the k-NN does not build an explicit mathematical model during training; it simply memorizes the entire training dataset and makes predictions for new and unseen data points by evaluating the neighbors of a query point. Because of this, the k-NN is often referred to as a lazy learning or instance-based algorithm.

The core idea of the k-NN model is that the object data points that are near each other in feature space are usually from the same category or have similar predicted values. Therefore, to estimate the label of a new input sample $x_{new}$, the k-NN model finds the k training points that are closest to $x_{new}$ and uses their labels (or values) to produce the prediction. The closest point is determined based on a distance metric, typically Euclidean distance, which is given by the following:

(18) $$d\left(x_{i},x_{j}\right)=\sqrt{\sum_{m=1}^{p}{{(x}_{im}-x_{jm)}}^{2}}$$

where $x_{i},x_{j}$ denote two data vectors and p denotes the number of features.

After identifying the k closest neighbors to a query point $x_{new}$, the prediction $y^{\prime }$ is obtained by averaging the corresponding target values $\left(y_{i}\right),$ as presented as follows:

(19) $$y^{\prime }=\frac{1}{k}\sum_{i=1}^{k}y_{i}$$

The best performance of the k-NN model was achieved with k = 3, Euclidean distance metric, inverse distance weighting rule and normalized input attributes.

#### 2.2.6. K-Star

The Random Tree (RT) model is a simplified version of RF, referring to a single tree constructed by incorporating random elements. To build an RT, the model first randomly selects a subset of the training data. At each splitting node of the RT, only a random subset of features is considered, rather than all features.

The K-Star is an instance-based ML model applicable to both regression and classification tasks. Unlike traditional instance-based learners like the k-NN models, which rely on geometric distances such as Euclidean distance, the K-Star algorithm employs a probabilistic distance function defined by the concept of entropy and information theory. The core idea of the K-Star algorithm is utilized to determine how much information is needed to transform one instance into another using a series of stochastic transitions. Because it uses a probability distribution rather than raw numerical differences, the K-Star is recognized to handle noise, missing values, categorical variables and non-linear relationships more effectively than the k-NN model. The K-Star determines a probabilistic distance $\left[K^{*}\left(x,y\right)\right]$ between two instances, x and y, using Equation (20).

(20) $$K^{*}\left(x,y\right)=-log{\;P}^{*}\left(y|x\right)$$

where $P^{*}\left(y|x\right)$ denotes a large probability, which is defined by summing all possible transformation paths τ from x and y, as follows:

(21) $${\;P}^{*}\left(y|x\right)=\sum_{t\epsilon T(x\to y)}P(\tau )$$

where T(x→y) denotes the set of all valid transformation sequences and P(τ) expresses the probability corresponding to a specific transformation sequence.

#### 2.2.7. Decision Tree

A decision tree is a supervised, non-parametric learning algorithm that can handle both classification and regression by modeling the target variable using decision rules learned from the features. The process is recursive and uses a top-down approach, resulting in the structure of a tree-like graph comprising a root node, followed by internal nodes and leaf nodes (terminal nodes). The root node expresses the entire dataset, the internal node expresses a feature test that splits the data into two or more homogeneous subsets, and the terminal nodes (referred to as leaf nodes) express the final decision or prediction, as graphically described in Figure 3. This structure makes the decision tree versatile, interpretable and capable of capturing non-linear relationships and feature interactions.

For the prediction task, the decision tree model aims to create nodes where the target variables are as similar as possible by minimizing the variance defined by the sum of squared errors, as expressed in Equation (22).

(22) $$SSE=\sum_{x_{i}\in L}{\left(y_{i}-{\bar{y}}_{L}\right)}^{2}+\sum_{x_{i}\in R}{\left(y_{i}-{\bar{y}}_{R}\right)}^{2}$$

where ${\bar{y}}_{L}$ denotes the mean of the node.

#### 2.2.8. M5 Tree

The M5 Tree is a model tree specially used for regression tasks. Unlike traditional decision trees, which produce a constant value at each leaf, an M5 Tree generates a multiple linear regression model at each leaf, thereby greatly improving the prediction accuracy of the continuous output. An M5 Tree is considered a hybrid approach by combining the high-capacity prediction of linear regression and the partitioning power of decision trees. This method enables the model to represent complex, non-linear relationships by using locally optimized linear functions in different regions of the feature space.

Similarly to the structure of the decision tree, the M5 Tree structure is a tree-like graph, which comprises the root node, followed by internal nodes and terminal nodes. The root and internal nodes of the M5 Tree have similar functions as those of the decision tree, which, respectively, represent the entire data and feature test, while the terminal nodes (leaves) contain multiple linear regression models, as given by the following:

(23) $$y^{\prime }=\omega_{0}+\omega_{1}x_{1}+\omega_{2}x_{2}+\cdots +\omega_{P}x_{P}$$

#### 2.2.9. M5Rules Models

M5Rules is a powerful rule-based regression algorithm derived from the M5 model Tree. Unlike the M5 model tree, which produces a tree structure, M5Rules generates an ordered list of “if-then” rules that often provide a more interpretable presentation of the underlying model. In other words, M5Rules transforms each path of an M5 model tree into a set of linear regression rules, allowing the model to retain the predictive accuracy of the M5 Tree models while achieving the simplicity and clarity associated with rule-based systems. An individual in an M5Rules model has the general form as follows:

(24) $$\mathrm{IF}\;C_{1}\cap C_{1}\cap \ldots C_{k}\;THEN\;y^{\prime }=\omega_{0}+\omega_{1}x_{1}+\omega_{2}x_{2}+\cdots +\omega_{P}x_{P}$$

where $C_{i}$ denotes the decision condition; k denotes the number of conditions in the rule; p denotes the number of input variables in the linear model and $\omega_{i}$ denotes regression coefficients estimated by least squares.

#### 2.2.10. Decision Table

The Decision Table is an instance-based or lazy learning method that operates on a simple principle: it stores the entire training dataset and utilizes it to predict for new instances by looking up the most similar entries in the table. Unlike Decision Trees, which employ a hierarchical structure to split the data, the Decision Table is organized into a table of conditions and corresponding decisions. Because of this, the Decision Table is considered one of the most straightforward and most interpretable models in ML.

A Decision Table usually has three main components, including the condition attributes (schema), condition values (tests) and decision entries (class labels or numeric value). For a training instance of x= $\left(x_{1},x_{2},\ldots ,x_{p}\right)$, by assuming the schema of S= $\left(A_{1},A_{2},\ldots ,A_{k}\right)$ as the selected subset of attributes, a rule of a Decision Table is defined as follows:

(25) $$R_{j}=\overset{k}{\underset{i=1}{⋀}}\left(A_{i}=v_{ij}\right)$$

The decision made by each rule is given by the following:

(26) $$d_{j}=\frac{1}{\left\lceil T_{j}\right\rceil }\sum_{x\in T_{j}}y(x)$$

where $T_{j}$ denotes the set of training instances matching the rule $R_{j}$.

#### 2.2.11. Ensemble Models

Ensemble models are a powerful ML technique that combines multiple individual models (called base learners) to solve a particular computational intelligence problem. The core idea is that a diverse set of models can capture different structures or patterns in the data, leading to more stable and accurate predictive performance. For the ensemble model to be effective, the errors made by the individual base learners must be uncorrelated. That means the combination of single ML models, which have the same error on the same data points, offers no advantage. Accordingly, the models are often trained using different subsets of data, feature subsets and algorithms.

The three common ensemble techniques are bagging, boosting and stacking, which rely on different strategies to construct and combine base learners. For M base learners of $h_{1}\left(x\right),$ $h_{2}\left(x\right),\ldots .,h_{M}\left(x\right)$, an ensemble model developed based on the base learners aims to produce a final predictor $y^{\prime }\left(x\right)$ and can be expressed as follows:

(27) $$y^{\prime }(x)=A(h_{1}\left(x\right),h_{2}\left(x\right),\;\ldots .,h_{M}\left(x\right))$$

where A(.) is a combination function such as averaging, voting or a weighted sum.

Bagging aims to reduce prediction variance by training multiple base learners on different random subsets of the training data. This process converts weak learners into strong learners by minimizing a global loss function. For a regression task, the final ensemble prediction $y^{\prime }(x)$ is typically the average of the predictions from all M base learners:

(28) $$y^{\prime }(x)=\frac{1}{M}\sum_{m=1}^{M}h_{m}\left(x\right)$$

Unlike bagging, boosting attempts to iteratively convert a set of weak learners into a single strong learner by running the models in parallel. Boosting trains models sequentially, with each new model attempting to correct the errors of the previous models. This technique assigns a weight to each learner based on its accuracy using Equation (24), then defines the final ensemble prediction $y^{\prime }(x)$ using Equation (30):

(29) $$\alpha_{m}=\frac{1}{2}ln(\frac{1-\in_{m}}{\in_{m}})$$

(30) $$y^{\prime }(x)=sign\left(\sum_{m=1}^{M}{\alpha_{m}h}_{m}\left(x\right)\right)$$

Stacking is the most complex technique of the ensemble model, integrating predictions from multiple diverse base models by training a meta-learner on their output. In the stacking method, a K-fold cross-validation is used on the training set to generate out-of-fold predictions so the meta-learner does not overfit. By assuming the predictions of based models are $z_{m}\left(x\right)={\alpha_{m}h}_{m}\left(x\right)$, a new dataset is constructed as follows:

(31) $$Z={\left\{z_{1}\left(x_{1}\right),\;z_{2}\left(x_{2}\right),\ldots ,z_{M}\left(x_{i}\right),\;y_{i}\right\}}_{i=1}^{N}$$

A trained meta-model $g\left(.\right)$ is used to determine the final ensemble prediction $y^{\prime }(x)$:

(32) $$y^{\prime }(x)=g(h_{1}\left(x\right),h_{2}\left(x\right),\;\ldots .,h_{M}\left(x\right))$$

#### 2.2.12. Model Construction and Ten-Fold Cross-Validation Technique

The selected ML models in this study were constructed using Weka (Waikato Environment for Knowledge Analysis), an open-source data mining and machine learning software. The default structures of the Weka ML models were first used, followed by a trial-and-error process to optimize the parameters adopted in the selected ML models.

In this study, a 10-fold cross-validation technique was used to assess the performance of the selected machine learning models using a dataset comprising 547 experimental results on the ultimate strain of FRP-CC columns. This validation technique is widely recognized for balancing computational efficiency and variance reduction [63]. Specifically, the dataset was randomly partitioned into 10 roughly equal subsets (folds), each representing about 10% of the total data. The cross-validation procedure involved 10 iterations; in each iteration, nine folds served as the training set and the remaining fold was used as the test set. Performance metrics for the models were calculated on the respective test fold in every iteration, and the final reported metrics were obtained by averaging these values across all 10 iterations.

## 3. Test Database

### 3.1. Data Collections

A test database consisting of 547 data points on the ultimate strain of FCC columns was collected from 70 experimental investigations available in the literature [20,58,64,65,66,67,68,69,70,71,72,73,74,75,76,77,78,79,80,81,82,83,84,85,86,87,88,89,90,91,92,93,94,95,96,97,98,99,100,101,102,103,104,105,106,107,108,109,110,111,112,113,114,115,116,117,118,119,120,121,122,123,124,125,126,127,128,129,130,131]. The test results of FCC columns satisfying the following conditions were included in this study: (1) the fiber direction of FRP-CC columns was perpendicular to the column axis; (2) the failure of FRP-CC columns was due to the rupture of the FRP jacket instead of the debonding of FRP materials; and (3) the aspect ratio (H/D) of FRP-CC columns was less than 3. The FCC columns assembled in this study were externally strengthened by different types of FRP materials, consisting of Aramid FRP, Glass FRP, Carbon FRP (CFRP) and High Modulus and Ultra High Modulus CFRP.

**Figure 4:**
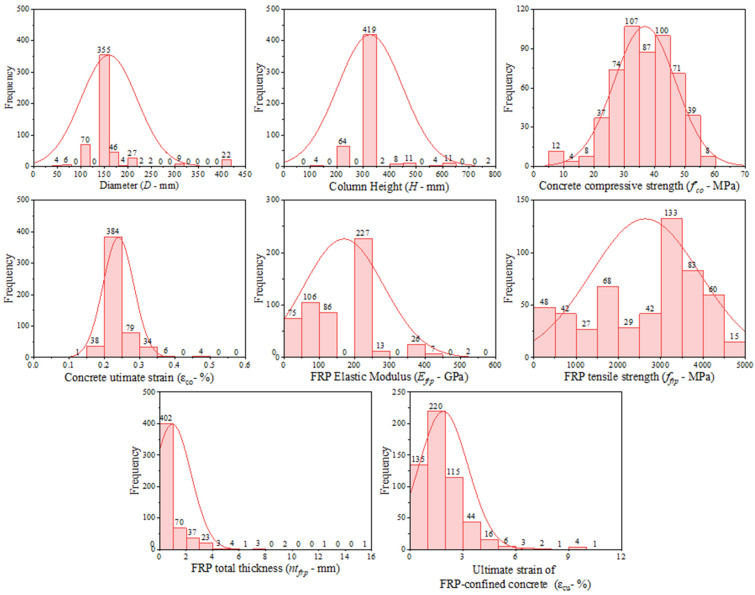
Histograms for input and output variables.

For the prediction of the ultimate axial strain of FRP-CC columns ($\varepsilon_{cu})$ using the AI models, the following input parameters were employed: (1) column height,H; (2) column diameter, D; (3) compressive strength of control concrete, $f_{co}$; (4) ultimate strain of control concrete, $\varepsilon_{co}$; (5) FRP elastic modulus, $E_{frp}$; (6) FRP tensile strength, $f_{frp}$, and (7) FRP total thickness, $nt_{frp}$. Accordingly, the ML models were trained and tested using seven input and one output parameters. Similar input parameters were used in Mozumder et al. [44] and Naderpour et al. [39] for estimating the strength capacity of FRP-CC columns. Most of these input parameters were adopted by Keshtegar et al. [49] for predicting the ultimate conditions of FRP-CC columns. In this study, the values of H and D varied from 50 mm to 406.4 mm and from 100 mm to 812.8 mm, respectively. The values of $f_{co}$ and $\varepsilon_{co}$ fell in the range of 6.2 MPa to 55.2 MPa and 0.14% to 0.63%, respectively. The values of $E_{frp}$, $f_{frp}$ and $nt_{frp}$, respectively, varied from 4.9 GPa to 640 GPa, from 75 MPa to 4510 MPa and from 0.057 mm to 15 mm. The value of $\varepsilon_{cu}$ varied from 0.23% to 10.4%. The statistical characteristics of the database are schematically presented in Figure 4. The details of the test database used for predicting the ultimate strain of FPR-CC columns were presented in Table S1 of the Supplementary Material.

### 3.2. Pearson’s Correlation Analysis

**Figure 5:**
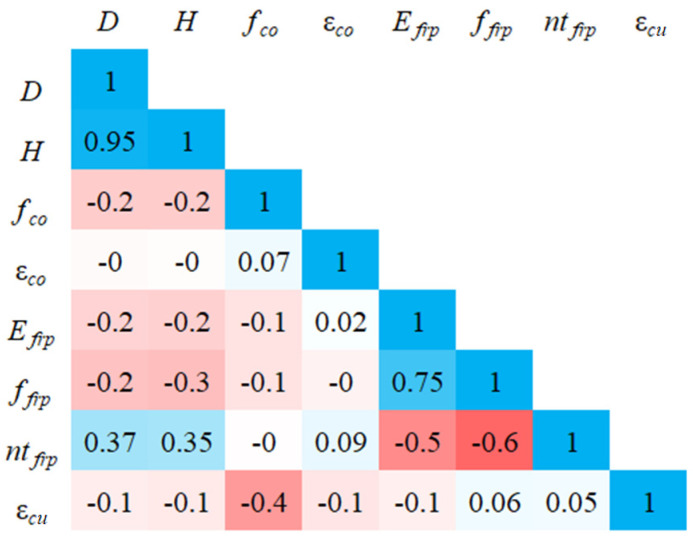
Heatmap of Pearson correlation coefficient between variables.

The correlation between the seven input and output variables was determined using the Pearson correlation coefficient, as presented in Figure 5. It can be seen from Figure 5 that FRP tensile strength and total thickness had a positive influence on the ultimate strain of the FRP-CC column, while the five remaining variables, including column diameter $\left(D\right)$ and height $\left(H\right)$, compressive strength $\left(f_{co}\right)$ and ultimate strain $\left(\varepsilon_{co}\right)$ of control concrete and FRP elastic modulus $\left(E_{frp}\right)$, had a negative influence on the ultimate strain of FRP-CC columns. It should be noted that the positive influence of FRP tensile strength $\left(f_{frp}\right)$ and total thickness $\left(nt_{frp}\right)$ was negligible, while the negative influence of the compressive strength of the control concrete $\left(f_{co}\right)$ was considerable. This indicated that there was no strong linear correlation between the input and output variables. In other words, the correlation between the input parameter and output parameter is non-linear and may be complex.

## 4. Results and Discussion

### 4.1. Statistical Indicators

The accuracy of ensemble ML models in estimating the strain capacity of FRP-CC columns was compared with that of the single ML and empirical models based on four statistical indicators of correlation coefficient (R), mean absolute error (MAE), root mean square error (RMSE), mean absolute percentage error (MAPE) and synthetic indicator (SI). The SI indicator was determined based on three statistical indicators of MAPE, RMSE and MAE. The statistical indicators employed in this study were given by the following:

(33) $$R=\frac{n\sum yy^{\prime }-(\sum y)(\sum y^{\prime })}{\left(\sqrt{n(\sum y^{2})-{\left(\sum y\right)}^{2}\;}\right)\sqrt{n((\sum y^{\prime 2})-{(\sum y^{\prime })}^{2}}}$$

(34) $$MAPE=\frac{1}{n}\sum_{1}^{n}\left|\frac{y-y^{\prime }}{y}\right|$$

(35) $$MSE=\sqrt{\frac{1}{n}\sum_{1}^{n}{\left(y-y^{\prime }\right)}^{2}}$$

(36) $$MAE=\frac{1}{n}\sum_{1}^{n}\left|y-y^{\prime }\right|$$

(37) $$SI=\frac{1}{m}\sum_{1}^{m}\left|\frac{P_{i}-P_{min,i}}{P_{max,i}-P_{min,i}}\right|$$

where $y^{\prime }$ and y denote the predicted and actual labels; n denotes the number of data points; $P_{i}$ denotes the ith statistical indicator; $P_{min,i}$ and $P_{max,i}$ denote the minimum and maximum values of the ith statistical indicator, respectively, m denotes the number of statistical indicators.

### 4.2. The Estimation Accuracy of the Models

#### 4.2.1. Performance of Empirical Strain and Single ML Models

**Figure 6:**
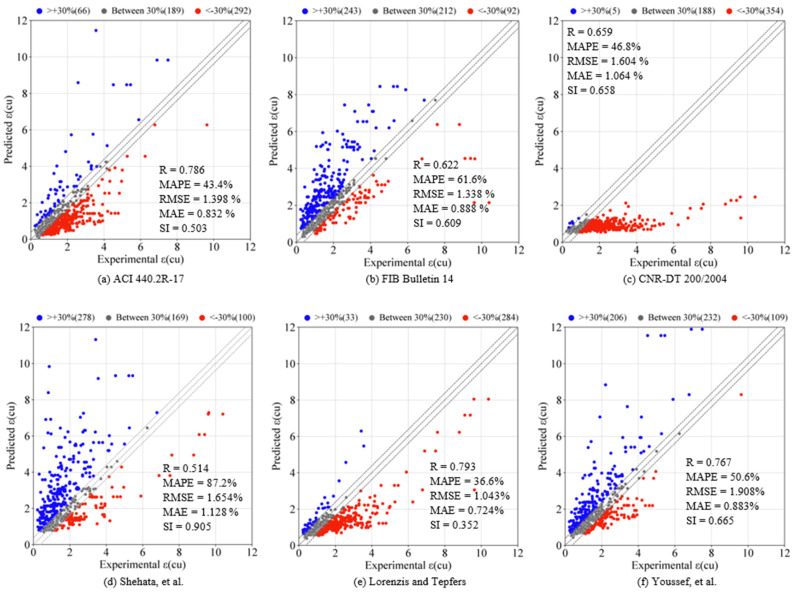

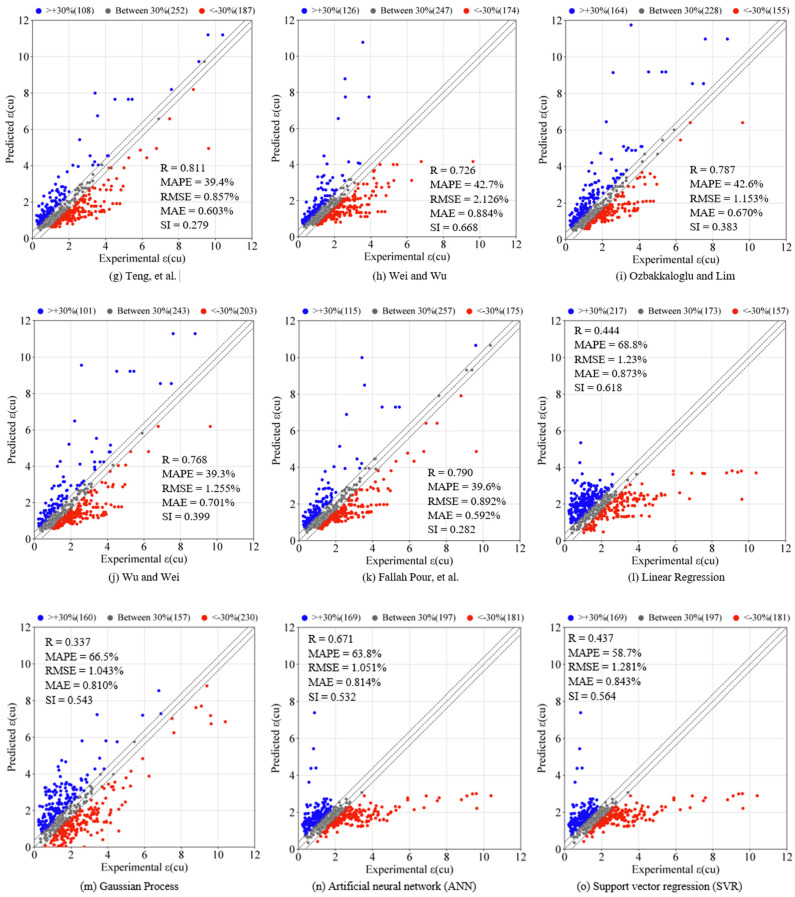

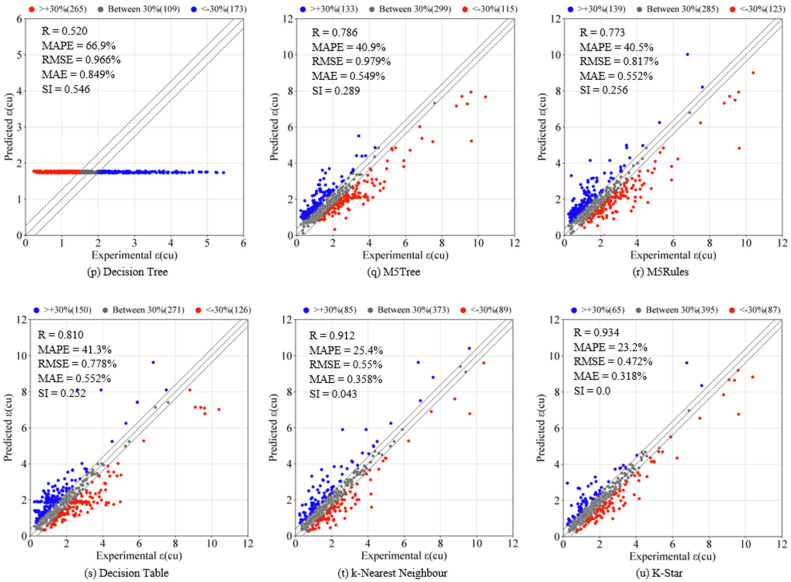
Predicted vs. experimental $\varepsilon_{cu}$ of FRP-CC columns.

**Figure 7:**
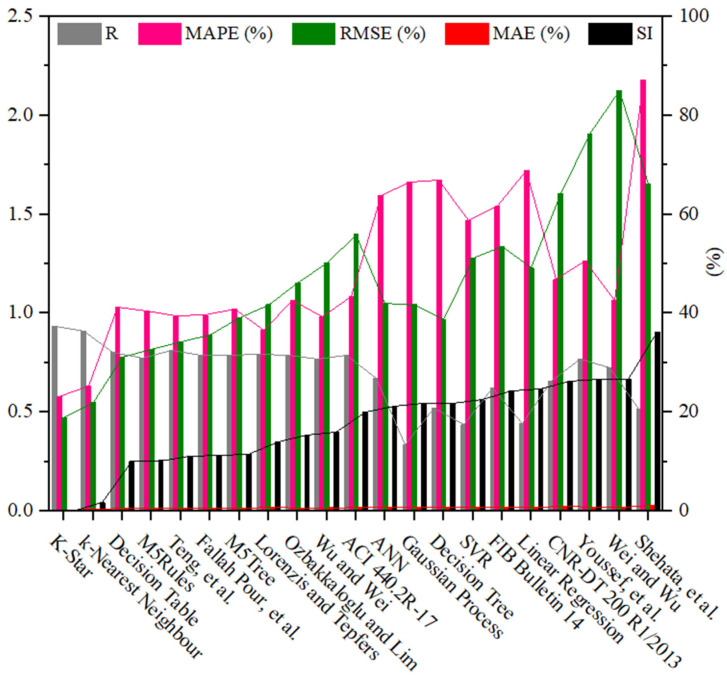
Comparison between the empirical and ML models in estimating the $\varepsilon_{cu}$ of FRP-CC columns using various indicators.

**Figure 8:**
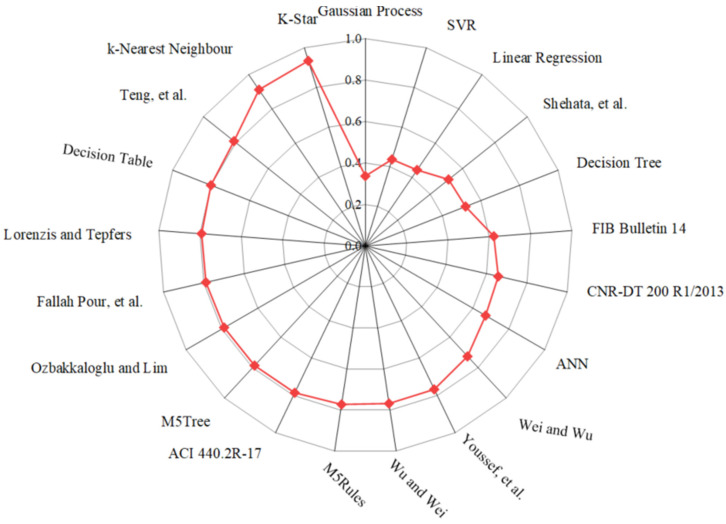
Comparison between the empirical and ML models in estimating the $\varepsilon_{cu}$ of FRP-CC columns using the correlation coefficient $\left(R\right)$ indicator.

**Figure 9:**
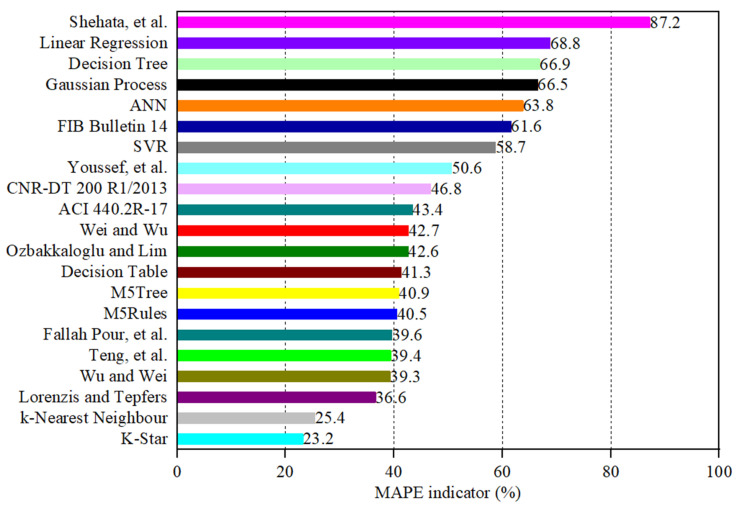
Comparison between the empirical and ML models in estimating the $\varepsilon_{cu}$ of FRP-CC columns using the mean absolute percentage error (MAPE) indicator.

**Figure 10:**
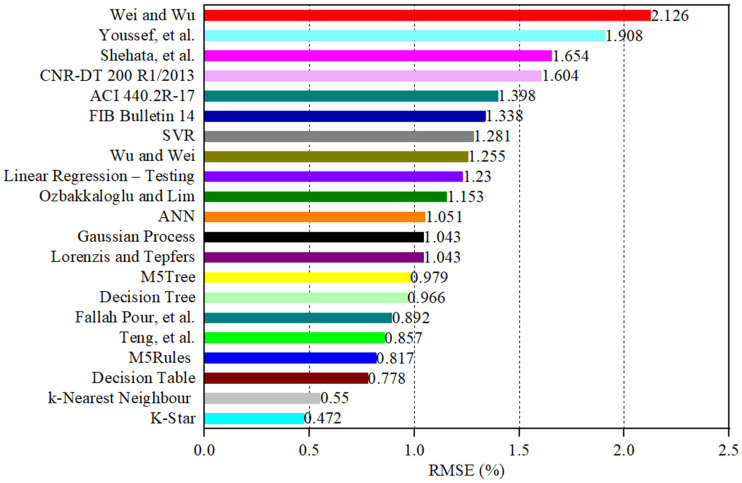
Comparison between the empirical and ML models in estimating the $\varepsilon_{cu}$ of FRP-CC columns using the root mean square error $\left(RMSE\right)$ indicator.

**Figure 11:**
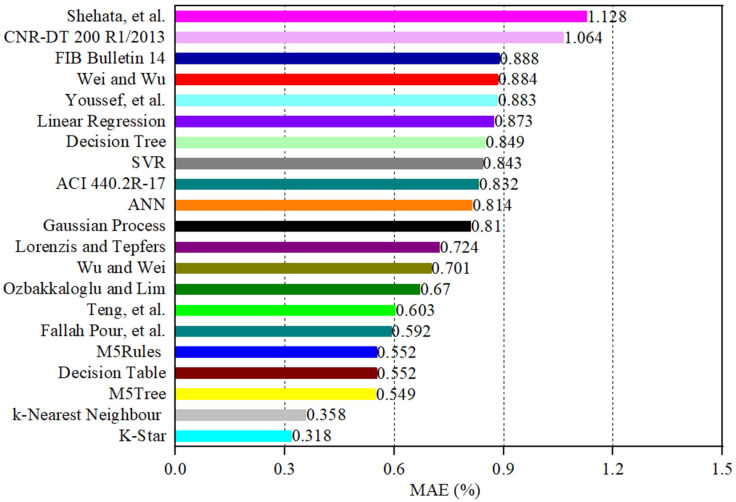
Comparison between the empirical and ML models in estimating the $\varepsilon_{cu}$ of FRP-CC columns using the mean absolute error $\left(MAE\right)$ indicator.

**Figure 12:**
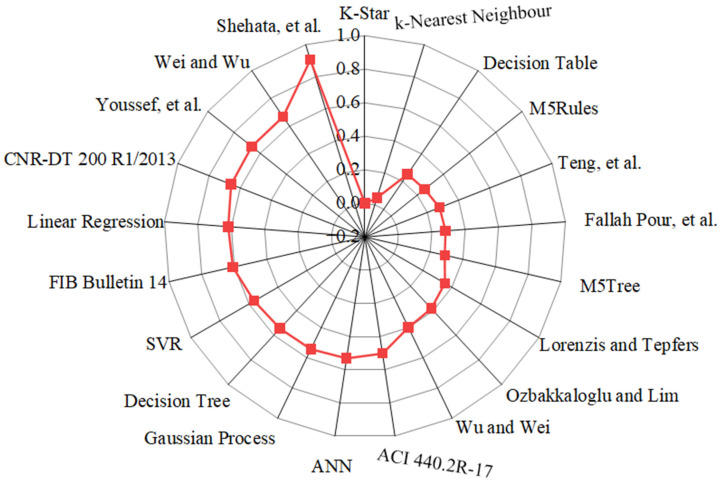
Comparison between empirical strength and ML models in estimating the $\varepsilon_{cu}$ of FRP-SCC columns using the SI.

The estimated values of $\varepsilon_{cu}$ obtained from the empirical strain and ML models are plotted against their actual values in Figure 6a–u. A comparison between the performance of the empirical and ML models using different statistical indicators is presented in Table 2 and graphically in Figure 7, Figure 8, Figure 9, Figure 10, Figure 11 and Figure 12. In Figure 6, the 450 diagonal line shows perfect agreement between the predicted value and the actual one, while the two diagonal lines (upper and lower threshold lines) parallel to the 450 diagonal line express the threshold of 30% difference between the estimated and actual values. For three strain models adopted in design codes of ACI 440.2R-17 [53], FIB Bulletin 14 [54] and CNR-DT 200/2004 [55], as illustrated in Figure 6, most data points presenting the correlation between the estimated and actual strain obtained by the CNR-DT 200/2004 [55] strain model were under the lower threshold line, indicating that the CNR-DT 200/2004 [55] design code underestimated the strain capacity of FRP-CC columns. In contrast, a large number of data points obtained from the FIB Bulletin 14 [54] strain model were distributed above the upper threshold line, revealing that the FIB Bulletin 14 [54] overestimated the $\varepsilon_{cu}$ of FRP-CC columns. It can also be found in Figure 6 that the distribution of data points obtained by Shehata et al. [56] was above the upper threshold line, which can be attributed to the fact that this empirical model overestimated the $\varepsilon_{cu}$ of FRP-CC columns.

It should be noted that a large number of data points obtained by Teng et al. [19] and Fallah Pour et al. [61] empirical strain models (252 and 257 data points, respectively) fell in the region formed by the upper and lower threshold lines (hereafter referred to as the threshold region), while the remaining data points were closely distributed along the upper and lower threshold lines, showing that these two empirical models effectively estimated the $\varepsilon_{cu}$ of the FRP-CC columns. It is interesting to find that the data points obtained by the decision tree model are distributed horizontally, indicating that this ML model provided an unsatisfactory prediction of the $\varepsilon_{cu}$ of FRP-CC columns. In contrast to the decision tree model, a large number of data points obtained by the K-Star, k-Nearest Neighbor, M5 Tree, M5Rules and Decision Table models (395, 373, 299, 285 and 271 data points, respectively) were located within the threshold region, showing that these single ML models yielded good estimations of the $\varepsilon_{cu}$ of FRP-CC columns.

As can also be seen in Table 2 and Figure 7, different statistical indicators resulted in distinct best strain models. However, the K-Star model consistently performed the best model, followed by the k-Nearest Neighbor model in estimating the $\varepsilon_{cu}$ of the FRP-CC columns. By using the R indicator, as indicated in Figure 7, the best empirical strain model was Teng et al.’s [19] strain model, followed by the Lorenzis and Tepfers [57] and Fallah Pour et al. [61] strain models. The empirical strain model having the lowest prediction accuracy was Shehata et al. [56], while the best ML models in predicting the $\varepsilon_{cu}$ of FRP-CC columns were K-Star, k-Nearest Neighbor and Decision Table models. The estimation accuracy of the K-Star and k-Nearest Neighbor models was 15.2% and 12.5% higher than that of the Teng et al. [19] strain model, while the estimation accuracy of the Decision Table model was 1.23% lower than that of the Teng et al. [19] strain model. For the use of the MAPE indicator, as shown in Figure 8, the best empirical strain models were Lorenzis and Tepfers [57], Wu and Wei [60] and Teng et al. [19], respectively, while the empirical strain model with the lowest prediction accuracy was Shehata et al. [56]. The K-Star and k-Nearest Neighbor models obtained 36.6% and 30.6% higher prediction accuracy than the Lorenzis and Tepfers [6] strain model. It should be noted that the MAPE indicators of the best ML models in this study exceeded 20%, as also observed by Keshtegar et al. [49], Lim et al. [50] and Mansouri et al. [51]. Interestingly, the MAPE indicators achieved by the two best ML models in this study were lower than those of the ML models adopted in Mansouri et al. [51] and the empirical models proposed by Keshtegar et al. [49] and Lim et al. [50] using ML models. For the use of the RMSE indicator, the best empirical strain model was the Teng et al. [19] strain model and the best single ML models were the K-Star, k-Nearest Neighbor, Decision Table models and M5Rules models. The increases in the estimation accuracy of these ML models in comparison to the Teng et al. [19] strain model were 44.9%, 35.8%, 9.2% and 4.7%, respectively. By using the MAE indicator, the best empirical model was the Fallah Pour et al. [61] strain model, which had a 46.3%, 39.5%, 7.3%, 6.8% and 6.8%, respectively, lower prediction accuracy than the best ML models of the K-Star, k-Nearest Neighbor, M5Tree, Decision Table and M5Rules models.

**Figure 13:**
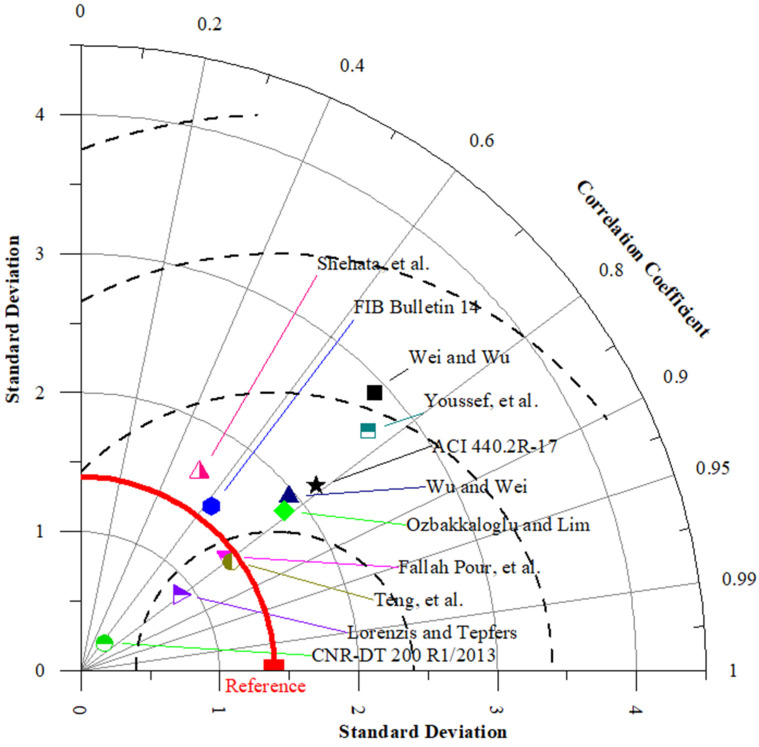
Taylor diagrams of typical empirical strain models applied to the testing data.

As different statistical indicators resulted in the distinct best strain models, the SI was employed to determine the best model for estimating the $\varepsilon_{cu}$ of FRP-CC columns. Taylor diagrams were also constructed to assess the best empirical strain models of FRP-CC columns, as illustrated in Figure 13. As can be seen in Figure 13, the best empirical strain model was achieved by the strain model proposed by Teng et al. [19], followed by the strain models proposed by Fallah Pour et al. [61], Lorenzis and Tepfers [6], Ozbakkaloglu and Lim [14] and Wu and Wei [60], while Shehata et al. [56] obtained the lowest prediction accuracy, which was followed by Wei and Wu [59] and Youssef et al. [58]. Notably, the best models obtained by the Taylor diagram were consistent with those received from the unified SI indicator, with the top empirical models being Teng et al. [19], followed by Fallah Pour et al. [61], Lorenzis and Tepfers [6], Ozbakkaloglu and Lim [14] and Wu and Wei [60]. However, by using the Taylor diagram, the Wei and Wu [59] strain model had the lowest prediction accuracy, followed by the Youssef et al. [58] strain model. It should be noted that, based on the SI, the four best single ML models in estimating the $\varepsilon_{cu}$ of FRP-CC columns were the K-Star, k-Nearest Neighbor, M5Tree, Decision Table and M5Rules models, respectively. It should be mentioned that, among the strain models adopted in three design codes for concrete externally strengthened by FRP materials, the ACI 440.2R-17 [53] strain model obtained the highest prediction accuracy, followed by the FIB Bulletin 14 [54] strain model, while CNR-DT 200 R1/2013 [55] had the lowest prediction accuracy in estimating the $\varepsilon_{cu}$ of FRP-CC columns.

#### 4.2.2. Performance of Ensemble ML Models

**Figure 14:**
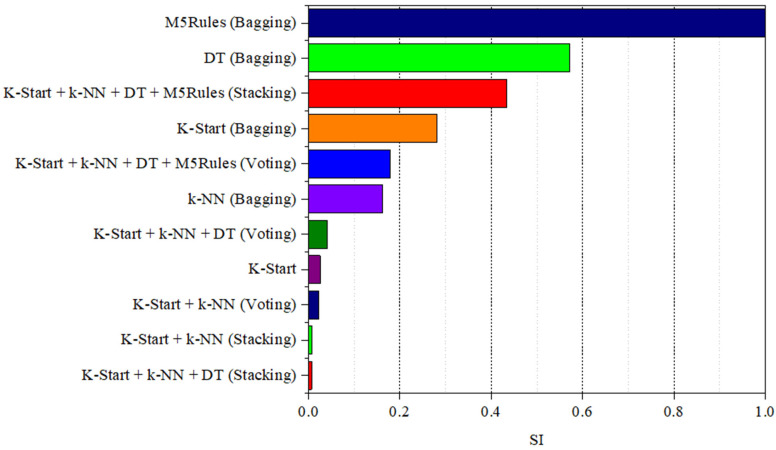
Comparison between ensemble and best single ML model in estimating the $\varepsilon_{cu}$ of FRP-CC columns using SI.

To investigate the estimation accuracy of ensemble ML models in estimating the $\varepsilon_{cu}$ of FRP-CC columns, the four best single ML models, consisting of K-Star, k-Nearest Neighbor, M5Tree, Decision Table and M5Rules models, were used to develop ensemble models based on the voting, stacking and bagging techniques. Table 3 indicates the performance of the developed ensemble models compared to the best single ML model of K-Star, while Figure 14 compares the performance of the ensemble models to the best single ML model. It is evident in Table 3 and Figure 14 that, by using the SI, the ensemble models developed employing the voting technique for the K-Star and k-Nearest Neighbor models, the stacking technique for the K-Star and k-Nearest Neighbor models and the stacking technique for the K-Star, k-Nearest Neighbor and Decision Table models performed better than the single K-Star model. The ensemble models with better indicators than the single K-Star model are shown in bold in Table 3. It should be noted that the voting-based ensemble model comprising K-Star and k-Nearest Neighbor obtained a better estimation accuracy than the K-Star model based on the MAPE indicator and the same estimation accuracy as the one based on the MAE indicator but had a lower prediction accuracy based on the R and RMSE indicators.

The estimation accuracy of the stacking-based ensemble model comprising K-Star, k-Nearest Neighbor and Decision Table models obtained the highest prediction accuracy, which was slightly higher than that of the K-Star model, based on R,MAPE, RMSE and MAE indicators; however, it was significantly better (74% higher) than that of the K-Star model, based on the SI. The second-best ensemble model was the stacking-based ensemble model comprising K-Star and k-Nearest Neighbor models, which had marginally better prediction accuracy than the K-Star model, based on R,MAPE, RMSE and MAE indicators, but remarkably higher prediction accuracy, based on the SI (73.2% higher). The voting-based ensemble model comprising K-Star and k-Nearest Neighbor models, which obtained the third-best prediction accuracy, had a 12% higher prediction accuracy than the K-Star model, based on the SI.

## 5. Concluding Remarks

This study comprehensively investigates the performance of ensemble ML models in estimating the strain capacity of FRP-CC columns. The performance of 10 single ML models was compared with that of 11 empirical strain models. The ensemble models were developed based on the four best single ML models. The findings of this paper are summarized as follows:

(1) Different statistical indicators resulted in distinct best strain models for FRP-CC columns. When assessed using a unified statistical indicator (SI), the empirical strain model developed by Teng et al. emerged as the most accurate, followed by those proposed by Fallah Pour et al. and by Lorenzis and Tepfers. Among the strain models specified in three design codes for concrete strengthened externally with FRP, the model from ACI 440.2R-17 provided the highest accuracy in predicting the strain capacity of FRP-CC columns, followed by the FIB Bulletin 14 model, whereas the CNR-DT 200 R1/2013 model exhibited the lowest accuracy.

(2) The single ML models provided good estimation for the strain capacity of FRP-CC columns. The best results were achieved by K-Star and k-Nearest Neighbor models. The estimation accuracies of the K-Star and k-Nearest Neighbor models were 15.2% and 12.5% higher, respectively, than those of the Teng et al. strain model based on the correlation coefficient (R) indicator. The increase in estimation accuracy in the K-Star and k-Nearest Neighbor models compared to Teng et al.’s strain model was 41.1% and 35.9% for the mean absolute percentage error (MAPE) indicator, respectively, 44.9% and 35.8% for the root mean square error (RMSE) indicator and 47.3% and 40.6% for the mean absolute error (MAE) indicator.

(3) K-Star and k-Nearest Neighbor models, the two best single ML models used in this study, achieved a better mean absolute percentage error (MAPE) indicator than other single ML models and empirical models based on ML models from the literature in estimating the strain capacity of FRP-CC columns.

(4) Among the ML models, the stacking-based ensemble model comprising K-Star, k-Nearest Neighbor and Decision Table achieved the highest prediction accuracy, followed by the stacking-based ensemble model comprising K-Star and k-Nearest Neighbor and the voting-based ensemble model comprising K-Star and k-Nearest Neighbor.

This study is limited to the performance of single and ensemble ML models in predicting the strain capacity of FRP-CC columns. Further investigations into the influence of input parameters on the accuracy of ML models in estimating the ultimate strain of FRP-CC columns are needed. In addition, the application of hybrid ML models in predicting the strain capacity of FRP-confined concrete columns should be further studied to improve the prediction accuracy of ML models.

## Figures and Tables

**Table 1:** Empirical models for the ultimate strain of FRP-confined concrete columns.

No	Reference	Compressive Strength Formulation
1	ACI 440.2R-17 [53]	$\frac{\varepsilon_{cu}}{\varepsilon_{co}}=1.5+12{\frac{f_{l}}{f_{co}}\left(\frac{\varepsilon_{fe}}{\varepsilon_{co}}\right)}^{0.45}$ $f_{l}=\frac{2E_{f}nt_{f}\varepsilon_{fe}}{D}$ $\varepsilon_{fe}=0.58\varepsilon_{fu}$
2	FIB Bulletin 14 [54]	$\frac{\varepsilon_{cu}}{\varepsilon_{co}}={\left[\frac{2\beta \varepsilon_{fu}E_{cc}}{E_{c}-E_{cc}}\right]}^{1-E_{cc}/E_{c}}$ $E_{cc}=\frac{f_{cc}^{\prime }}{\varepsilon_{cc}}$ $\frac{\varepsilon_{cc}}{\varepsilon_{co}}=1+5\left(\frac{f_{cc}^{\prime }}{f_{co}}-1\right)$ $\frac{f_{cc}^{\prime }}{f_{co}}=2.254{\left(1+7.94\frac{f_{lu}}{f_{co}}\right)}^{0.5}-2\frac{f_{lu}}{f_{co}}-1.254$ $\beta =\frac{5700}{\sqrt{\left\lceil f_{co}\right\rceil }}-500$ $f_{lu}=\frac{2E_{f}nt_{f}\varepsilon_{fu}}{D}$
3	CNR-DT 200 R1/2013 [55]	$\varepsilon_{cu}=0.0035+0.015\sqrt{\frac{f_{l}}{f_{co}}}$ $f_{l}=\frac{2E_{f}nt_{f}\varepsilon_{fe}}{D}$ $\varepsilon_{fe}\;$ $=\min\;\left\{\;\eta_{a}\varepsilon_{fu}/\gamma_{f};0.004\right\}$
4	Shehata et al. [56]	$\frac{\varepsilon_{cu}}{\varepsilon_{co}}=1+632{\left(\frac{f_{lu}}{f_{co}}.\frac{f_{cc}}{E_{f}}\right)}^{0.5}$ $f_{lu}=\frac{2E_{f}nt_{f}\varepsilon_{fu}}{D}$
5	Lorenzis and Tepfers [57]	$\frac{\varepsilon_{cu}}{\varepsilon_{co}}=1+26.2{\left(\frac{f_{lu}}{f_{co}}\right)}^{0.8}{E_{l}}^{-0.148}$ $E_{l}=\frac{2E_{f}t_{f}}{D}$
6	Youssef et al. [58]	$\varepsilon_{cu}=0.003368+0.2590{\frac{f_{lu}}{f_{co}}\left(\frac{f_{fu}}{E_{f}}\right)}^{0.5}$ $f_{lu}=\frac{2E_{f}nt_{f}\varepsilon_{fu}}{D}$
7	Teng et al. [19]	$\frac{\varepsilon_{cu}}{\varepsilon_{co}}=1.75+6.5\rho_{K}^{0.8}\rho_{\varepsilon }^{1.45}$ $\rho_{k}=\frac{{2E}_{f}t_{f}}{D(f_{co}/\varepsilon_{co})}$ $\rho_{\varepsilon }=\frac{\varepsilon_{fe}}{\varepsilon_{co}}$ $\varepsilon_{fe}=0.586\varepsilon_{fu}$
8	Wei and Wu [59]	$\frac{\varepsilon_{cu}}{\varepsilon_{co}}=1.75+12{\left(\frac{f_{lu}}{f_{co}}\right)}^{0.75}{\left(\frac{f_{30}}{f_{co}}\right)}^{0.62}$ $f_{lu}=\frac{2E_{f}nt_{f}\varepsilon_{fu}}{D}$
9	Ozbakkaloglu and Lim [14]	$\varepsilon_{cu}=c_{2}\varepsilon_{co}+0.27{\left(\frac{K_{l}}{f_{co}}\right)}^{0.9}\varepsilon_{fe}^{1.35}$ $c_{2}=2-\frac{f_{co}-20}{100}\geq 1$ $\varepsilon_{co}=\left(-0.067f_{co}^{2}+29.9f_{co}+1053\right)\times 10^{-6}$ $K_{l}=\frac{{2E}_{f}t_{f}}{D}\geq f_{co}^{1.65}$ $\varepsilon_{fe}=k_{\varepsilon }\varepsilon_{fu}$ $k_{\varepsilon }=0.9-2.3*f_{co}\times 10^{-3}-0.75*E_{f}\times 10^{-6}$
10	Wu and Wei [60]	$\frac{\varepsilon_{cu}}{\varepsilon_{co}}=1.75+140\left(\frac{f_{lu}}{f_{co}}\right)\varepsilon_{fu}^{0.6}$ $f_{lu}=\frac{2E_{f}t_{f}\varepsilon_{fu}}{D}$
11	Fallah Pour et al. [61]	$\varepsilon_{cu}=1.5\varepsilon_{co}+k_{2}{\left(\frac{K_{l}}{f_{co}}\right)}^{0.75}\varepsilon_{fu}^{1.35}$ $K_{l}=\frac{{2E}_{f}t_{f}}{D}$ $k_{2}=0.3-0.001f_{co}$

**Table 2:** Statistical indicators for strain estimation $\left(\varepsilon_{cu}\right).$

No.	Model	Indicator				
		R	MAPE (%)	RMSE (%)	MAE (%)	SI
1	ACI 440.2R-17 [53]	0.786	43.4	1.398	0.832	0.503
2	FIB Bulletin 14 [54]	0.622	61.6	1.338	0.888	0.609
3	CNR-DT 200 R1/2013 [55]	0.659	46.8	1.604	1.064	0.658
4	Shehata et al. [56]	0.514	87.2	1.654	1.128	0.905
5	Lorenzis and Tepfers [57]	0.793	36.6	1.043	0.724	0.352
6	Youssef et al. [58]	0.767	50.6	1.908	0.883	0.665
7	Teng et al. [19]	0.811	39.4	0.857	0.603	0.279
8	Wei and Wu [59]	0.726	42.7	2.126	0.884	0.668
9	Ozbakkaloglu and Lim [14]	0.787	42.6	1.153	0.670	0.383
10	Wu and Wei [60]	0.768	39.3	1.255	0.701	0.399
11	Fallah Pour et al. [61]	0.790	39.6	0.892	0.592	0.283
12	Linear Regression—Testing	0.444	68.8	1.23	0.873	0.619
	Linear Regression—(Training)	(0.484)	(66.8)	(1.22)	(0.852)	
13	Gaussian Process—Testing	0.337	66.5	1.043	0.810	0.543
	Gaussian Process—(Training)	(0.397)	(67.4)	(1.310)	(0.886)	
14	ANN—Testing	0.671	63.8	1.051	0.814	0.532
	ANN—(Training)	(0.786)	(57.4)	(0.928)	(0.725)	
15	SVR—Testing	0.437	58.7	1.281	0.843	0.564
	SVR—(Training)	(0.469)	(57.3)	(1.302)	(0.828)	
16	Decision Tree—Testing	0.520	66.9	0.966	0.849	0.546
	Decision Tree—(Training)	(0.589)	(71.3)	(1.149)	(0.761)	
17	M5Tree—Testing	0.786	40.9	0.979	0.549	0.289
	M5Tree—(Training)	(0.895)	(33.1)	(0.639)	(0.452)	
18	M5Rules—Testing	0.773	40.5	0.817	0.552	0.255
	M5Rules—Training	(0.890)	(35.2)	(0.637)	(0.460)	
19	Decision Table—Testing	0.801	41.3	0.778	0.552	0.252
	Decision Table—(Training)	(0.890)	(27.3)	(0.634)	(0.396)	
20	k-Nearest Neighbor—Testing	0.912	25.4	0.550	0.358	0.043
	k-Nearest Neighbor—(Training)	(0.993)	(4.4)	(0.166)	(0.079)	
21	K-Star—Testing	0.934	23.2	0.472	0.318	0
	K-Star—(Training)	(0.991)	(6.13)	(0.199)	(0.110)	

**Table 3:** Statistical indicators of ensemble models for strain estimation.

No	Model		Indicators				
			R	MAPE (%)	RMSE (%)	MAE (%)	SI
Original	K-Star	**Testing**	**0.934**	**23.2**	**0.472**	**0.318**	**0.0250**
		Training	0.991	6.13	0.199	0.110	
Voting	K-Star + k-NN	**Testing**	0.932	**22.9**	0.475	**0.318**	**0.0220**
		Training	0.992	5.2	0.175	0.092	
	K-Star + k-NN + DT	Testing	0.932	23.0	0.478	0.325	0.0396
		Training	0.984	11.1	0.259	0.168	
	K-Star + k-NN + DT + M5Rules	Testing	0.923	25.2	0.522	0.348	0.1785
		Training	0.979	15.1	0.317	0.215	
Stacking	K-Star + k-NN	**Testing**	**0.935**	**23.1**	**0.467**	**0.312**	**0.0067**
		Training	0.992	5.9	0.180	0.099	
	K-Star + k-NN + DT	**Testing**	**0.936**	**23.2**	**0.465**	**0.312**	**0.0065**
		Training	0.990	7.6	0.197	0.120	
	K-Star + k-NN + DT + M5Rules	Testing	0.887	28.4	0.591	0.409	0.4332
		Testing	0.950	19.0	0.448	0.282	
Bagging	K-Star	Testing	0.905	27.0	0.551	0.366	0.2825
		Training	0.981	12.4	0.276	0.172	
	k-NN	Testing	0.927	25.1	0.508	0.349	0.1615
		Training	0.980	12.7	0.302	0.189	
	DT	Testing	0.883	31.7	0.619	0.429	0.5721
		Training	0.944	23.6	0.478	0.318	
	M5Rules	Testing	0.833	38.2	0.748	0.508	1.00
		Training	0.884	32.7	0.668	0.434	

## Data Availability

The original contributions presented in this study are included in the article/supplementary material. Further inquiries can be directed to the corresponding author.

## Supplementary Materials

The following supporting information can be downloaded at https://www.mdpi.com/article/10.3390/ma19010189/s1, Table S1: Test database for prediction of ultimate strain of FRP-CC columns.
