# Supplementary material for: Investigation of Ensemble Machine Learning Models for Estimating the Ultimate Strain of FRP-Confined Concrete Columns
Source: Materials (Basel). 2026 Jan 4;19(1):189. doi: 10.3390/ma19010189 (PMC12787090; doi:10.3390/ma19010189)
Supplement: Supplementary file 1 [file materials-19-00189-s001.zip › materials-4068324-supplementary.pdf]

# Investigation of Ensemble Machine Learning Models for Estimating the Ultimate Strain of FRP-Confined Concrete Columns

**Quang Trung Nguyen [1], Anh Duc Pham [1], Quynh Chau Truong [1], Cong Luyen Nguyen [2], Ngoc Son Truong [1] and Anh Duc Mai [1,*]**

[1] Construction Management Division, The University of Da Nang—University of Science and Technology, Da Nang 550000, Vietnam; nqtrung@dut.udn.vn (Q.T.N.); paduc@dut.udn.vn (A.D.P.); tqchau@dut.udn.vn (Q.C.T.); tnson@dut.udn.vn (N.S.T.)
[2] Construction Informatics Division, The University of Da Nang—University of Science and Technology, Da Nang 550000, Vietnam; ncluyen@dut.udn.vn
* Correspondence: maduc@dut.udn.vn

Table S1. Test database for estimation of ultimate strain of FRP-CC columns

| X1 | X2 | X3 | X4 | X5 | X6 | X7 | Y |
|---|---|---|---|---|---|---|---|
| 150.00 | 300.00 | 42.00 | 0.24 | 240.00 | 3900.00 | 0.117 | 0.92 |
| 150.00 | 300.00 | 42.00 | 0.24 | 240.00 | 3900.00 | 0.351 | 2.12 |
| 150.00 | 300.00 | 42.00 | 0.24 | 240.00 | 3900.00 | 0.702 | 3.16 |
| 100.00 | 200.00 | 26.5 | 0.31 | 242.00 | 3248.00 | 0.167 | 2.55 |
| 100.00 | 200.00 | 26.5 | 0.31 | 242.00 | 3248.00 | 0.167 | 2.18 |
| 100.00 | 200.00 | 26.5 | 0.31 | 242.00 | 3248.00 | 0.167 | 2.29 |
| 100.00 | 200.00 | 26.5 | 0.31 | 242.00 | 3248.00 | 0.167 | 2.48 |
| 200.00 | 400.00 | 21.7 | 0.22 | 242.00 | 3248.00 | 0.334 | 2.79 |
| 200.00 | 400.00 | 21.7 | 0.22 | 242.00 | 3248.00 | 0.334 | 2.69 |
| 200.00 | 400.00 | 21.7 | 0.22 | 242.00 | 3248.00 | 0.334 | 2.10 |
| 200.00 | 400.00 | 21.7 | 0.22 | 242.00 | 3248.00 | 0.334 | 2.54 |
| 300.00 | 600.00 | 24.5 | 0.22 | 242.00 | 3248.00 | 0.501 | 1.80 |
| 300.00 | 600.00 | 24.5 | 0.22 | 242.00 | 3248.00 | 0.501 | 2.00 |
| 300.00 | 600.00 | 24.5 | 0.22 | 242.00 | 3248.00 | 0.501 | 1.90 |
| 300.00 | 600.00 | 24.5 | 0.22 | 242.00 | 3248.00 | 0.501 | 2.00 |
| 150.00 | 300.00 | 32.4 | 0.205 | 75.1 | 935.00 | 1.2 | 3.23 |
| 150.00 | 300.00 | 36.2 | 0.205 | 75.1 | 935.00 | 1.2 | 3.23 |
| 160.00 | 320.00 | 25.9 | 0.273 | 238.00 | 4300.00 | 0.13 | 1.28 |
| 160.00 | 320.00 | 25.9 | 0.273 | 238.00 | 4300.00 | 0.39 | 1.52 |
| 160.00 | 320.00 | 49.5 | 0.169 | 238.00 | 4300.00 | 0.13 | 0.25 |
| 160.00 | 320.00 | 49.5 | 0.169 | 238.00 | 4300.00 | 0.39 | 0.73 |
| 160.00 | 320.00 | 25.0 | 0.233 | 230.00 | 3200.00 | 0.165 | 1.63 |
| 160.00 | 320.00 | 25.0 | 0.233 | 230.00 | 3200.00 | 0.165 | 0.93 |
| 160.00 | 320.00 | 25.0 | 0.233 | 230.00 | 3200.00 | 0.165 | 1.67 |
| 160.00 | 320.00 | 25.0 | 0.233 | 230.00 | 3200.00 | 0.330 | 1.73 |
| 160.00 | 320.00 | 25.0 | 0.233 | 230.00 | 3200.00 | 0.330 | 1.58 |
| 160.00 | 320.00 | 25.0 | 0.233 | 230.00 | 3200.00 | 0.330 | 1.68 |
| 160.00 | 320.00 | 40.1 | 0.200 | 230.00 | 3200.00 | 0.110 | 0.55 |
| 160.00 | 320.00 | 40.1 | 0.200 | 230.00 | 3200.00 | 0.110 | 0.66 |
| 160.00 | 320.00 | 40.1 | 0.200 | 230.00 | 3200.00 | 0.110 | 0.61 |
| 160.00 | 320.00 | 40.1 | 0.200 | 230.00 | 3200.00 | 0.165 | 0.66 |
| 160.00 | 320.00 | 40.1 | 0.200 | 230.00 | 3200.00 | 0.165 | 0.62 |
| 160.00 | 320.00 | 40.1 | 0.200 | 230.00 | 3200.00 | 0.165 | 0.64 |

| | | | | | | | |
|---|---|---|---|---|---|---|---|
| 160.00 | 320.00 | 40.1 | 0.200 | 230.00 | 3200.00 | 0.220 | 0.60 |
| 160.00 | 320.00 | 40.1 | 0.200 | 230.00 | 3200.00 | 0.220 | 0.69 |
| 160.00 | 320.00 | 40.1 | 0.200 | 230.00 | 3200.00 | 0.220 | 0.73 |
| 160.00 | 320.00 | 40.1 | 0.200 | 230.00 | 3200.00 | 0.440 | 1.44 |
| 160.00 | 320.00 | 40.1 | 0.200 | 230.00 | 3200.00 | 0.440 | 1.36 |
| 160.00 | 320.00 | 40.1 | 0.200 | 230.00 | 3200.00 | 0.440 | 1.17 |
| 160.00 | 320.00 | 40.1 | 0.200 | 230.00 | 3200.00 | 0.990 | 2.46 |
| 160.00 | 320.00 | 40.1 | 0.200 | 230.00 | 3200.00 | 0.990 | 2.39 |
| 160.00 | 320.00 | 40.1 | 0.200 | 230.00 | 3200.00 | 1.32 | 2.70 |
| 160.00 | 320.00 | 52.0 | 0.227 | 230.00 | 3200.00 | 0.330 | 0.83 |
| 160.00 | 320.00 | 52.0 | 0.227 | 230.00 | 3200.00 | 0.330 | 0.70 |
| 160.00 | 320.00 | 52.0 | 0.227 | 230.00 | 3200.00 | 0.330 | 0.77 |
| 160.00 | 320.00 | 52.0 | 0.227 | 230.00 | 3200.00 | 0.660 | 1.14 |
| 160.00 | 320.00 | 52.0 | 0.227 | 230.00 | 3200.00 | 0.660 | 1.12 |
| 160.00 | 320.00 | 52.0 | 0.227 | 230.00 | 3200.00 | 0.660 | 1.12 |
| 150.00 | 300.00 | 34.4 | 0.33 | 231.00 | 4100.00 | 0.12 | 0.80 |
| 150.00 | 300.00 | 34.4 | 0.33 | 231.00 | 4100.00 | 0.12 | 0.87 |
| 150.00 | 300.00 | 34.4 | 0.33 | 231.00 | 4100.00 | 0.12 | 0.90 |
| 100.00 | 200.00 | 28.0 | 0.25 | 231.00 | 4100.00 | 0.12 | 1.32 |
| 100.00 | 200.00 | 28.0 | 0.25 | 231.00 | 4100.00 | 0.12 | 1.06 |
| 100.00 | 200.00 | 20.1 | 0.207 | 230.00 | 3430.00 | 0.165 | 2.55 |
| 254.00 | 762.00 | 38.9 | 0.30 | 72.50 | 875.00 | 1.0 | 1.04 |
| 152.00 | 305.00 | 33.5 | 0.23 | 25.00 | 350.00 | 1.7 | 0.93 |
| 152.00 | 305.00 | 48.1 | 0.222 | 85.00 | 816.00 | 1.00 | 1.53 |
| 152.00 | 305.00 | 48.1 | 0.222 | 85.00 | 816.00 | 2.00 | 2.01 |
| 152.00 | 305.00 | 48.1 | 0.222 | 85.00 | 816.00 | 2.00 | 2.66 |
| 152.00 | 305.00 | 48.1 | 0.222 | 85.00 | 816.00 | 3.00 | 3.09 |
| 152.00 | 305.00 | 48.1 | 0.222 | 85.00 | 816.00 | 3.00 | 2.89 |
| 152.00 | 305.00 | 45.6 | 0.247 | 241.00 | 3639.00 | 0.11 | 1.21 |
| 152.00 | 305.00 | 45.6 | 0.247 | 241.00 | 3639.00 | 0.11 | 1.31 |
| 152.00 | 305.00 | 45.6 | 0.247 | 241.00 | 3639.00 | 0.22 | 1.97 |
| 152.00 | 305.00 | 45.6 | 0.247 | 241.00 | 3639.00 | 0.22 | 2.14 |
| 152.00 | 305.00 | 45.6 | 0.247 | 241.00 | 3639.00 | 0.33 | 2.90 |
| 152.00 | 305.00 | 45.6 | 0.247 | 241.00 | 3639.00 | 0.33 | 2.83 |
| 152.00 | 305.00 | 48.1 | 0.222 | 85.00 | 816.00 | 1.00 | 1.51 |
| 152.00 | 305.00 | 32.2 | 0.22 | 25.00 | 380.00 | 1.00 | 1.41 |
| 152.00 | 305.00 | 43.7 | 0.24 | 25.00 | 380.00 | 1.00 | 0.97 |
| 152.00 | 305.00 | 43.7 | 0.24 | 25.00 | 380.00 | 3.00 | 1.83 |
| 152.00 | 305.00 | 43.7 | 0.24 | 25.00 | 380.00 | 3.00 | 1.83 |
| 50.00 | 100.00 | 53.8 | 0.344 | 77.3 | 846.00 | 1.00 | 1.56 |
| 100.00 | 200.00 | 49.1 | 0.361 | 77.3 | 846.00 | 1.00 | 1.09 |
| 100.00 | 200.00 | 49.1 | 0.361 | 77.3 | 846.00 | 2.00 | 1.54 |
| 150.00 | 300.00 | 41.1 | 0.362 | 77.3 | 846.00 | 1.00 | 0.95 |
| 150.00 | 300.00 | 41.1 | 0.362 | 77.3 | 846.00 | 2.00 | 1.34 |
| 150.00 | 300.00 | 41.1 | 0.362 | 77.3 | 846.00 | 3.00 | 1.49 |
| 150.00 | 300.00 | 11.1 | 0.3 | 230.00 | 3430.00 | 0.165 | 4.20 |
| 150.00 | 300.00 | 20.8 | 0.3 | 230.00 | 3430.00 | 0.165 | 3.50 |
| 152.00 | 305.00 | 37.3 | 0.23 | 240.00 | 3800.00 | 0.234 | 1.31 |
| 51.00 | 102.00 | 41.00 | 0.24 | 235.00 | 3500.00 | 0.179 | 1.10 |

| | | | | | | | |
|---|---|---|---|---|---|---|---|
| 51.00 | 102.00 | 41.00 | 0.24 | 235.00 | 3500.00 | 0.344 | 2.00 |
| 51.00 | 102.00 | 41.00 | 0.24 | 235.00 | 3500.00 | 0.690 | 3.40 |
| 152.00 | 305.00 | 32.1 | 0.28 | 25.00 | 350.00 | 1.00 | 0.60 |
| 152.00 | 305.00 | 32.1 | 0.28 | 25.00 | 350.00 | 2.00 | 0.86 |
| 152.00 | 305.00 | 32.1 | 0.28 | 25.00 | 350.00 | 3.00 | 1.38 |
| 200.00 | 600.00 | 41.7 | 0.34 | 230.00 | 3481.00 | 0.444 | 2.10 |
| 150.00 | 300.00 | 32.00 | 0.2 | 230.00 | 3430.00 | 0.165 | 1.44 |
| 150.00 | 300.00 | 32.00 | 0.2 | 230.00 | 3430.00 | 0.495 | 3.43 |
| 150.00 | 300.00 | 32.00 | 0.2 | 230.00 | 3430.00 | 0.495 | 3.92 |
| 150.00 | 300.00 | 32.00 | 0.2 | 230.00 | 3430.00 | 0.825 | 4.96 |
| 150.00 | 300.00 | 32.00 | 0.2 | 230.00 | 3430.00 | 0.825 | 4.32 |
| 150.00 | 300.00 | 6.2 | 0.2 | 230.00 | 3430.00 | 0.165 | 3.90 |
| 150.00 | 300.00 | 6.2 | 0.2 | 230.00 | 3430.00 | 0.165 | 2.60 |
| 150.00 | 300.00 | 6.2 | 0.2 | 230.00 | 3430.00 | 0.330 | 5.90 |
| 150.00 | 300.00 | 6.2 | 0.2 | 230.00 | 3430.00 | 0.330 | 5.90 |
| 150.00 | 300.00 | 6.2 | 0.2 | 230.00 | 3430.00 | 0.495 | 6.90 |
| 150.00 | 300.00 | 6.2 | 0.2 | 230.00 | 3430.00 | 0.495 | 7.50 |
| 150.00 | 300.00 | 6.2 | 0.2 | 230.00 | 3430.00 | 0.660 | 8.80 |
| 150.00 | 300.00 | 6.2 | 0.2 | 230.00 | 3430.00 | 0.660 | 7.60 |
| 150.00 | 300.00 | 6.2 | 0.2 | 230.00 | 3430.00 | 0.825 | 9.10 |
| 150.00 | 300.00 | 6.2 | 0.2 | 230.00 | 3430.00 | 0.825 | 9.40 |
| 150.00 | 300.00 | 6.2 | 0.2 | 230.00 | 3430.00 | 0.990 | 10.40 |
| 150.00 | 300.00 | 6.2 | 0.2 | 230.00 | 3430.00 | 0.990 | 9.60 |
| 200.00 | 320.00 | 38.5 | 0.276 | 240.00 | 3720.00 | 0.117 | 0.80 |
| 200.00 | 320.00 | 38.5 | 0.276 | 240.00 | 3720.00 | 0.117 | 0.71 |
| 200.00 | 320.00 | 38.5 | 0.276 | 240.00 | 3720.00 | 0.117 | 0.35 |
| 200.00 | 320.00 | 38.5 | 0.276 | 240.00 | 3720.00 | 0.234 | 0.88 |
| 200.00 | 320.00 | 38.5 | 0.276 | 240.00 | 3720.00 | 0.234 | 0.58 |
| 200.00 | 320.00 | 38.5 | 0.276 | 240.00 | 3720.00 | 0.234 | 0.86 |
| 200.00 | 320.00 | 38.5 | 0.276 | 240.00 | 3720.00 | 0.351 | 1.76 |
| 200.00 | 320.00 | 38.5 | 0.276 | 240.00 | 3720.00 | 0.117 | 0.86 |
| 200.00 | 320.00 | 38.5 | 0.276 | 240.00 | 3720.00 | 0.117 | 1.24 |
| 200.00 | 320.00 | 35.7 | 0.191 | 240.00 | 3720.00 | 0.117 | 0.30 |
| 200.00 | 320.00 | 35.7 | 0.191 | 240.00 | 3720.00 | 0.234 | 0.60 |
| 200.00 | 320.00 | 35.7 | 0.191 | 240.00 | 3720.00 | 0.234 | 1.04 |
| 200.00 | 320.00 | 35.7 | 0.191 | 240.00 | 3720.00 | 0.234 | 1.07 |
| 200.00 | 320.00 | 35.7 | 0.191 | 240.00 | 3720.00 | 0.351 | 1.72 |
| 200.00 | 320.00 | 35.7 | 0.191 | 240.00 | 3720.00 | 0.351 | 1.71 |
| 200.00 | 320.00 | 35.7 | 0.191 | 240.00 | 3720.00 | 0.351 | 1.69 |
| 150.00 | 300.00 | 12.8 | 0.47 | 231.00 | 3650.00 | 0.12 | 1.37 |
| 150.00 | 300.00 | 12.8 | 0.47 | 231.00 | 3650.00 | 0.24 | 2.78 |
| 200.00 | 350.00 | 12.1 | 0.22 | 230.00 | 3500.00 | 0.12 | 1.92 |
| 152.00 | 305.00 | 38.4 | 0.23 | 138.1 | 1047.00 | 0.66 | 1.30 |
| 152.00 | 305.00 | 38.4 | 0.23 | 77.39 | 1105.00 | 0.99 | 2.20 |
| 152.00 | 305.00 | 38.4 | 0.23 | 95.7 | 1352.00 | 1.32 | 2.40 |
| 100.00 | 200.00 | 34.3 | 0.23 | 235.00 | 3820.00 | 0.167 | 0.79 |
| 100.00 | 200.00 | 34.3 | 0.23 | 235.00 | 3820.00 | 0.167 | 1.11 |
| 100.00 | 200.00 | 32.3 | 0.22 | 235.00 | 3820.00 | 0.167 | 1.07 |
| 100.00 | 200.00 | 32.3 | 0.22 | 235.00 | 3820.00 | 0.167 | 1.07 |

| | | | | | | | |
|---|---|---|---|---|---|---|---|
| 100.00 | 200.00 | 32.3 | 0.22 | 235.00 | 3820.00 | 0.334 | 1.75 |
| 100.00 | 200.00 | 32.3 | 0.22 | 235.00 | 3820.00 | 0.501 | 1.65 |
| 100.00 | 200.00 | 32.3 | 0.22 | 235.00 | 3820.00 | 0.501 | 1.59 |
| 100.00 | 200.00 | 34.8 | 0.23 | 235.00 | 3820.00 | 0.167 | 0.94 |
| 100.00 | 200.00 | 34.8 | 0.23 | 235.00 | 3820.00 | 0.167 | 1.05 |
| 100.00 | 200.00 | 34.8 | 0.23 | 235.00 | 3820.00 | 0.167 | 0.98 |
| 100.00 | 200.00 | 34.8 | 0.23 | 235.00 | 3820.00 | 0.334 | 2.06 |
| 100.00 | 200.00 | 34.8 | 0.23 | 235.00 | 3820.00 | 0.501 | 2.36 |
| 100.00 | 200.00 | 34.8 | 0.23 | 235.00 | 3820.00 | 0.501 | 2.49 |
| 152.00 | 305.00 | 35.9 | 0.203 | 230.00 | 3420.00 | 0.165 | 1.27 |
| 152.00 | 305.00 | 35.9 | 0.203 | 230.00 | 3420.00 | 0.165 | 1.11 |
| 152.00 | 305.00 | 35.9 | 0.203 | 230.00 | 3420.00 | 0.165 | 1.29 |
| 152.00 | 305.00 | 35.9 | 0.203 | 230.00 | 3420.00 | 0.330 | 1.68 |
| 152.00 | 305.00 | 35.9 | 0.203 | 230.00 | 3420.00 | 0.330 | 1.96 |
| 152.00 | 305.00 | 35.9 | 0.203 | 230.00 | 3420.00 | 0.330 | 1.85 |
| 152.00 | 305.00 | 34.3 | 0.19 | 230.00 | 3420.00 | 0.495 | 2.05 |
| 152.00 | 305.00 | 34.3 | 0.19 | 230.00 | 3420.00 | 0.495 | 2.41 |
| 152.00 | 305.00 | 34.3 | 0.19 | 230.00 | 3420.00 | 0.495 | 2.52 |
| 152.00 | 305.00 | 34.3 | 0.19 | 230.00 | 3420.00 | 0.165 | 1.02 |
| 152.00 | 305.00 | 34.3 | 0.19 | 230.00 | 3420.00 | 0.165 | 1.08 |
| 152.00 | 305.00 | 34.3 | 0.19 | 230.00 | 3420.00 | 0.165 | 1.17 |
| 150.00 | 300.00 | 36.2 | 0.24 | 250.00 | 4510.00 | 0.11 | 1.00 |
| 150.00 | 300.00 | 36.2 | 0.24 | 250.00 | 4510.00 | 0.22 | 1.50 |
| 150.00 | 300.00 | 36.2 | 0.24 | 250.00 | 4510.00 | 0.33 | 2.00 |
| 150.00 | 300.00 | 36.2 | 0.24 | 250.00 | 4510.00 | 0.44 | 2.70 |
| 150.00 | 300.00 | 36.2 | 0.24 | 250.00 | 4510.00 | 0.55 | 3.10 |
| 100.00 | 200.00 | 25.9 | 0.24 | 242.00 | 3591.00 | 0.167 | 2.31 |
| 100.00 | 200.00 | 25.9 | 0.24 | 242.00 | 3591.00 | 0.167 | 1.93 |
| 100.00 | 200.00 | 25.9 | 0.24 | 242.00 | 3591.00 | 0.167 | 2.16 |
| 100.00 | 200.00 | 25.9 | 0.24 | 242.00 | 3591.00 | 0.167 | 2.16 |
| 200.00 | 400.00 | 22.7 | 0.22 | 242.00 | 3591.00 | 0.334 | 2.29 |
| 200.00 | 400.00 | 22.7 | 0.22 | 242.00 | 3591.00 | 0.334 | 2.37 |
| 200.00 | 400.00 | 22.7 | 0.22 | 242.00 | 3591.00 | 0.334 | 2.00 |
| 200.00 | 400.00 | 22.7 | 0.22 | 242.00 | 3591.00 | 0.334 | 2.48 |
| 300.00 | 600.00 | 24.5 | 0.22 | 242.00 | 3591.00 | 0.501 | 1.84 |
| 300.00 | 600.00 | 24.5 | 0.22 | 242.00 | 3591.00 | 0.501 | 1.71 |
| 300.00 | 600.00 | 24.5 | 0.22 | 242.00 | 3591.00 | 0.501 | 2.27 |
| 300.00 | 600.00 | 24.5 | 0.22 | 242.00 | 3591.00 | 0.501 | 2.09 |
| 102.00 | 200.00 | 30.7 | 0.27 | 47.00 | 784.00 | 0.8 | 3.08 |
| 102.00 | 200.00 | 46.3 | 0.23 | 47.00 | 784.00 | 0.8 | 1.84 |
| 102.00 | 200.00 | 54.5 | 0.24 | 47.00 | 784.00 | 0.8 | 0.80 |
| 150.00 | 300.00 | 34.9 | 0.23 | 240.00 | 3900.00 | 0.117 | 0.85 |
| 102.00 | 204.00 | 37.0 | 0.23 | 227.00 | 3790.00 | 0.16 | 1.02 |
| 150.00 | 300.00 | 31.2 | 0.195 | 230.5 | 3481.00 | 0.11 | 1.21 |
| 150.00 | 300.00 | 31.2 | 0.195 | 230.5 | 3481.00 | 0.22 | 1.55 |
| 150.00 | 300.00 | 31.2 | 0.195 | 230.5 | 3481.00 | 0.33 | 2.01 |
| 100.00 | 200.00 | 33.7 | 0.190 | 230.5 | 3481.00 | 0.11 | 1.41 |
| 100.00 | 200.00 | 33.7 | 0.190 | 230.5 | 3481.00 | 0.22 | 1.49 |
| 100.00 | 200.00 | 33.7 | 0.190 | 230.5 | 3481.00 | 0.33 | 1.90 |

| | | | | | | | |
|---|---|---|---|---|---|---|---|
| 150.00 | 300.00 | 45.2 | 0.219 | 230.5 | 3481.00 | 0.11 | 0.95 |
| 150.00 | 300.00 | 45.2 | 0.219 | 230.5 | 3481.00 | 0.22 | 1.25 |
| 100.00 | 200.00 | 51.9 | 0.192 | 230.5 | 3481.00 | 0.11 | 0.96 |
| 100.00 | 200.00 | 51.9 | 0.192 | 230.5 | 3481.00 | 0.22 | 1.28 |
| 150.00 | 300.00 | 23.6 | 0.18 | 230.5 | 3481.00 | 0.11 | 1.59 |
| 150.00 | 300.00 | 23.6 | 0.18 | 230.5 | 3481.00 | 0.22 | 2.38 |
| 100.00 | 200.00 | 26.3 | 0.193 | 230.5 | 3481.00 | 0.11 | 1.99 |
| 100.00 | 200.00 | 26.3 | 0.193 | 230.5 | 3481.00 | 0.22 | 2.36 |
| 150.00 | 300.00 | 28.4 | 0.49 | 221.00 | 3430.00 | 0.165 | 2.20 |
| 150.00 | 300.00 | 38.2 | 0.63 | 221.00 | 3430.00 | 0.165 | 1.49 |
| 102.00 | 203.00 | 53.00 | 0.25 | 262.00 | 4200.00 | 0.165 | 1.00 |
| 102.00 | 203.00 | 53.00 | 0.25 | 262.00 | 4200.00 | 0.33 | 1.82 |
| 102.00 | 203.00 | 53.00 | 0.25 | 262.00 | 4200.00 | 0.66 | 2.32 |
| 102.00 | 203.00 | 53.00 | 0.25 | 262.00 | 4200.00 | 0.99 | 3.30 |
| 102.00 | 203.00 | 53.00 | 0.25 | 262.00 | 4200.00 | 1.32 | 4.17 |
| 152.00 | 305.00 | 47.9 | 0.25 | 262.00 | 4200.00 | 0.165 | 0.90 |
| 152.00 | 305.00 | 47.9 | 0.25 | 262.00 | 4200.00 | 0.33 | 1.69 |
| 152.00 | 305.00 | 47.9 | 0.25 | 262.00 | 4200.00 | 0.66 | 2.04 |
| 152.00 | 304.00 | 39.7 | 0.24 | 230.00 | 3400.00 | 0.33 | 1.07 |
| 100.00 | 200.00 | 42.00 | 0.24 | 230.00 | 3400.00 | 0.22 | 1.60 |
| 100.00 | 200.00 | 42.00 | 0.24 | 230.00 | 3400.00 | 0.22 | 1.57 |
| 100.00 | 200.00 | 42.00 | 0.24 | 230.00 | 3400.00 | 0.22 | 1.35 |
| 150.00 | 300.00 | 20.4 | 0.26 | 234.00 | 4493.00 | 0.17 | 0.96 |
| 150.00 | 300.00 | 20.4 | 0.26 | 234.00 | 4493.00 | 0.34 | 1.42 |
| 150.00 | 300.00 | 20.4 | 0.26 | 234.00 | 4493.00 | 0.51 | 1.42 |
| 150.00 | 300.00 | 49.2 | 0.17 | 234.00 | 4493.00 | 0.17 | 0.39 |
| 150.00 | 300.00 | 49.2 | 0.17 | 234.00 | 4493.00 | 0.34 | 0.35 |
| 150.00 | 300.00 | 49.2 | 0.17 | 234.00 | 4493.00 | 0.51 | 0.62 |
| 152.00 | 304.00 | 41.8 | 0.24 | 86.80 | 1220.00 | 1.00 | 1.18 |
| 152.00 | 304.00 | 47.5 | 0.25 | 86.80 | 1220.00 | 1.00 | 0.88 |
| 152.00 | 304.00 | 40.3 | 0.24 | 86.80 | 1220.00 | 2.00 | 2.04 |
| 152.00 | 304.00 | 41.7 | 0.24 | 86.80 | 1220.00 | 2.00 | 1.76 |
| 150.00 | 300.00 | 15.3 | 0.19 | 230.00 | 3400.00 | 0.11 | 0.45 |
| 150.00 | 300.00 | 15.3 | 0.19 | 230.00 | 3400.00 | 0.22 | 1.30 |
| 152.5 | 305.00 | 19.4 | 0.33 | 207.00 | 3654.00 | 0.5 | 1.59 |
| 152.5 | 305.00 | 19.4 | 0.33 | 207.00 | 3654.00 | 1.00 | 2.21 |
| 152.5 | 305.00 | 19.4 | 0.33 | 207.00 | 3654.00 | 1.5 | 2.58 |
| 152.5 | 305.00 | 19.4 | 0.33 | 207.00 | 3654.00 | 2.00 | 3.56 |
| 152.5 | 305.00 | 19.4 | 0.33 | 207.00 | 3654.00 | 2.5 | 3.42 |
| 152.5 | 305.00 | 49.00 | 0.29 | 207.00 | 3654.00 | 0.5 | 0.62 |
| 152.5 | 305.00 | 49.00 | 0.29 | 207.00 | 3654.00 | 1.00 | 0.97 |
| 152.5 | 305.00 | 49.00 | 0.29 | 207.00 | 3654.00 | 1.5 | 1.26 |
| 152.5 | 305.00 | 49.00 | 0.29 | 207.00 | 3654.00 | 2.00 | 1.90 |
| 150.00 | 300.00 | 29.8 | 0.21 | 235.00 | 3550.00 | 0.165 | 1.23 |
| 150.00 | 300.00 | 29.8 | 0.21 | 235.00 | 3550.00 | 0.330 | 1.74 |
| 225.00 | 450.00 | 34.00 | 0.2 | 235.00 | 3550.00 | 0.165 | 0.62 |
| 225.00 | 450.00 | 34.00 | 0.2 | 235.00 | 3550.00 | 0.330 | 1.09 |
| 150.00 | 300.00 | 34.00 | 0.2 | 235.00 | 3550.00 | 0.165 | 0.91 |
| 150.00 | 300.00 | 34.00 | 0.2 | 235.00 | 3550.00 | 0.330 | 1.10 |

| | | | | | | | |
|---|---|---|---|---|---|---|---|
| 100.00 | 300.00 | 22.4 | 0.20 | 237.00 | 4073.00 | 0.13 | 0.90 |
| 100.00 | 300.00 | 22.4 | 0.20 | 237.00 | 4073.00 | 0.26 | 1.76 |
| 100.00 | 300.00 | 22.4 | 0.20 | 237.00 | 4073.00 | 0.39 | 3.31 |
| 150.00 | 450.00 | 22.4 | 0.20 | 237.00 | 4073.00 | 0.13 | 1.22 |
| 150.00 | 450.00 | 22.4 | 0.20 | 237.00 | 4073.00 | 0.26 | 2.00 |
| 150.00 | 450.00 | 22.4 | 0.20 | 237.00 | 4073.00 | 0.39 | 2.56 |
| 100.00 | 300.00 | 40.9 | 0.24 | 237.00 | 4073.00 | 0.13 | 1.98 |
| 100.00 | 300.00 | 40.9 | 0.24 | 237.00 | 4073.00 | 0.26 | 1.65 |
| 100.00 | 300.00 | 40.9 | 0.24 | 237.00 | 4073.00 | 0.39 | 2.18 |
| 150.00 | 450.00 | 40.9 | 0.24 | 237.00 | 4073.00 | 0.13 | 0.87 |
| 150.00 | 450.00 | 40.9 | 0.24 | 237.00 | 4073.00 | 0.26 | 1.42 |
| 150.00 | 450.00 | 40.9 | 0.24 | 237.00 | 4073.00 | 0.39 | 1.89 |
| 152.5 | 305.00 | 49.0 | 0.25 | 238.00 | 4200.00 | 0.165 | 1.00 |
| 152.5 | 305.00 | 49.0 | 0.25 | 238.00 | 4200.00 | 0.330 | 1.80 |
| 152.5 | 305.00 | 49.0 | 0.25 | 238.00 | 4200.00 | 0.660 | 2.30 |
| 152.5 | 305.00 | 49.0 | 0.25 | 238.00 | 4200.00 | 0.990 | 3.70 |
| 152.5 | 305.00 | 49.0 | 0.25 | 238.00 | 4200.00 | 1.32 | 4.60 |
| 150.00 | 300.00 | 44.7 | 0.24 | 240.00 | 3800.00 | 0.234 | 0.86 |
| 150.00 | 300.00 | 20.8 | 0.241 | 234.00 | 4500.00 | 0.34 | 1.08 |
| 150.00 | 300.00 | 20.8 | 0.241 | 234.00 | 4500.00 | 0.34 | 1.32 |
| 150.00 | 300.00 | 48.8 | 0.251 | 234.00 | 4500.00 | 0.34 | 0.81 |
| 150.00 | 300.00 | 48.8 | 0.25 | 234.00 | 4500.00 | 0.34 | 0.90 |
| 150.00 | 300.00 | 40.0 | 0.17 | 234.00 | 4500.00 | 0.17 | 0.63 |
| 150.00 | 300.00 | 40.0 | 0.17 | 234.00 | 4500.00 | 0.34 | 1.07 |
| 150.00 | 300.00 | 40.0 | 0.17 | 234.00 | 4500.00 | 0.51 | 1.36 |
| 150.00 | 300.00 | 44.3 | 0.17 | 234.00 | 4500.00 | 0.17 | 0.58 |
| 150.00 | 300.00 | 44.3 | 0.17 | 234.00 | 4500.00 | 0.34 | 0.54 |
| 150.00 | 300.00 | 44.3 | 0.17 | 234.00 | 4500.00 | 0.51 | 0.94 |
| 152.00 | 305.00 | 35.5 | 0.23 | 240.00 | 3800.00 | 0.117 | 0.77 |
| 152.00 | 305.00 | 35.5 | 0.23 | 240.00 | 3800.00 | 0.117 | 0.82 |
| 152.00 | 305.00 | 35.5 | 0.23 | 240.00 | 3800.00 | 0.117 | 0.82 |
| 152.00 | 305.00 | 38.0 | 0.23 | 240.00 | 3800.00 | 0.234 | 1.51 |
| 152.00 | 305.00 | 38.0 | 0.23 | 240.00 | 3800.00 | 0.234 | 1.65 |
| 152.00 | 305.00 | 36.1 | 0.23 | 240.00 | 3800.00 | 0.234 | 1.27 |
| 100.00 | 200.00 | 30.2 | 0.22 | 235.00 | 3432.00 | 0.167 | 1.51 |
| 100.00 | 200.00 | 30.2 | 0.22 | 235.00 | 3432.00 | 0.501 | 3.11 |
| 100.00 | 200.00 | 30.2 | 0.22 | 235.00 | 3432.00 | 0.668 | 4.15 |
| 150.00 | 300.00 | 28.7 | 0.22 | 230.00 | 3400.00 | 0.167 | 2.53 |
| 150.00 | 300.00 | 28.7 | 0.22 | 230.00 | 3400.00 | 0.167 | 2.14 |
| 150.00 | 300.00 | 30.1 | 0.22 | 230.00 | 3400.00 | 0.334 | 3.89 |
| 150.00 | 300.00 | 30.1 | 0.22 | 230.00 | 3400.00 | 0.334 | 3.80 |
| 150.00 | 300.00 | 23.1 | 0.26 | 230.00 | 3400.00 | 0.167 | 2.01 |
| 150.00 | 300.00 | 23.1 | 0.26 | 230.00 | 3400.00 | 0.167 | 2.01 |
| 152.00 | 305.00 | 33.7 | 0.23 | 105.00 | 1577.00 | 0.381 | 1.20 |
| 152.00 | 305.00 | 33.7 | 0.23 | 105.00 | 1577.00 | 0.381 | 1.40 |
| 152.00 | 305.00 | 33.7 | 0.23 | 105.00 | 1577.00 | 0.381 | 1.24 |
| 152.00 | 305.00 | 33.7 | 0.23 | 105.00 | 1577.00 | 0.762 | 1.65 |
| 152.00 | 305.00 | 33.7 | 0.23 | 105.00 | 1577.00 | 0.762 | 2.25 |
| 152.00 | 305.00 | 33.7 | 0.23 | 105.00 | 1577.00 | 0.762 | 2.16 |

| | | | | | | | |
|---|---|---|---|---|---|---|---|
| 152.00 | 305.00 | 33.7 | 0.23 | 105.00 | 1577.00 | 1.14 | 2.45 |
| 152.00 | 305.00 | 33.7 | 0.23 | 105.00 | 1577.00 | 1.14 | 3.03 |
| 152.00 | 305.00 | 43.8 | 0.24 | 105.00 | 1577.00 | 0.381 | 0.98 |
| 152.00 | 305.00 | 43.8 | 0.24 | 105.00 | 1577.00 | 0.381 | 0.47 |
| 152.00 | 305.00 | 43.8 | 0.24 | 105.00 | 1577.00 | 0.381 | 0.37 |
| 152.00 | 305.00 | 43.8 | 0.24 | 105.00 | 1577.00 | 0.762 | 1.57 |
| 152.00 | 305.00 | 43.8 | 0.24 | 105.00 | 1577.00 | 0.762 | 1.37 |
| 152.00 | 305.00 | 43.8 | 0.24 | 105.00 | 1577.00 | 0.762 | 1.66 |
| 152.00 | 305.00 | 43.8 | 0.24 | 105.00 | 1577.00 | 1.14 | 1.74 |
| 152.00 | 305.00 | 43.8 | 0.24 | 105.00 | 1577.00 | 1.14 | 1.68 |
| 152.00 | 305.00 | 43.8 | 0.24 | 105.00 | 1577.00 | 1.14 | 1.75 |
| 152.00 | 305.00 | 55.2 | 0.26 | 105.00 | 1577.00 | 0.381 | 0.69 |
| 152.00 | 305.00 | 55.2 | 0.26 | 105.00 | 1577.00 | 0.381 | 0.48 |
| 152.00 | 305.00 | 55.2 | 0.26 | 105.00 | 1577.00 | 0.381 | 0.49 |
| 152.00 | 305.00 | 55.2 | 0.26 | 105.00 | 1577.00 | 0.762 | 1.21 |
| 152.00 | 305.00 | 55.2 | 0.26 | 105.00 | 1577.00 | 0.762 | 0.81 |
| 152.00 | 305.00 | 55.2 | 0.26 | 105.00 | 1577.00 | 1.14 | 1.43 |
| 152.00 | 305.00 | 55.2 | 0.26 | 105.00 | 1577.00 | 1.14 | 1.45 |
| 152.00 | 305.00 | 55.2 | 0.26 | 105.00 | 1577.00 | 1.14 | 1.18 |
| 305.00 | 610.00 | 15.0 | 0.20 | 86.90 | 1220.00 | 1.00 | 1.10 |
| 406.4 | 812.8 | 29.4 | 0.24 | 103.84 | 1246.00 | 5.84 | 2.81 |
| 406.4 | 812.8 | 29.4 | 0.24 | 103.84 | 1246.00 | 5.84 | 2.91 |
| 406.4 | 812.8 | 29.4 | 0.24 | 103.84 | 1246.00 | 5.84 | 2.80 |
| 406.4 | 812.8 | 29.4 | 0.24 | 103.84 | 1246.00 | 3.50 | 1.49 |
| 406.4 | 812.8 | 29.4 | 0.24 | 103.84 | 1246.00 | 3.50 | 1.62 |
| 406.4 | 812.8 | 29.4 | 0.24 | 103.84 | 1246.00 | 2.34 | 1.16 |
| 406.4 | 812.8 | 29.4 | 0.24 | 103.84 | 1246.00 | 2.34 | 1.11 |
| 406.4 | 812.8 | 29.4 | 0.24 | 103.84 | 1246.00 | 2.34 | 1.20 |
| 406.4 | 812.8 | 29.4 | 0.24 | 103.84 | 1246.00 | 1.17 | 0.65 |
| 406.4 | 812.8 | 29.4 | 0.24 | 103.84 | 1246.00 | 1.17 | 0.62 |
| 152.4 | 304.8 | 44.6 | 0.20 | 103.84 | 1246.00 | 2.34 | 2.85 |
| 152.4 | 304.8 | 44.6 | 0.20 | 103.84 | 1246.00 | 2.34 | 2.79 |
| 152.4 | 304.8 | 44.6 | 0.20 | 103.84 | 1246.00 | 2.34 | 2.84 |
| 152.4 | 304.8 | 44.6 | 0.20 | 103.84 | 1246.00 | 1.75 | 2.00 |
| 152.4 | 304.8 | 44.6 | 0.20 | 103.84 | 1246.00 | 1.75 | 2.00 |
| 152.4 | 304.8 | 44.6 | 0.20 | 103.84 | 1246.00 | 1.75 | 1.98 |
| 152.4 | 304.8 | 44.6 | 0.20 | 103.84 | 1246.00 | 1.17 | 1.71 |
| 152.4 | 304.8 | 44.6 | 0.20 | 103.84 | 1246.00 | 1.17 | 2.00 |
| 152.4 | 304.8 | 44.6 | 0.20 | 103.84 | 1246.00 | 1.17 | 2.00 |
| 150.00 | 300.00 | 34.3 | 0.23 | 240.00 | 3800.00 | 0.33 | 2.10 |
| 150.00 | 300.00 | 42.00 | 0.24 | 65.00 | 3000.00 | 0.15 | 0.73 |
| 150.00 | 300.00 | 42.00 | 0.24 | 65.00 | 3000.00 | 0.45 | 1.74 |
| 150.00 | 300.00 | 42.00 | 0.24 | 65.00 | 3000.00 | 0.894 | 2.50 |
| 150.00 | 300.00 | 47.7 | 0.308 | 27.00 | 540.00 | 1.3 | 1.49 |
| 150.00 | 300.00 | 47.7 | 0.308 | 27.00 | 540.00 | 3.9 | 2.72 |
| 150.00 | 300.00 | 50.8 | 0.294 | 27.00 | 540.00 | 1.3 | 0.97 |
| 150.00 | 300.00 | 50.8 | 0.294 | 27.00 | 540.00 | 3.9 | 0.97 |
| 150.00 | 375.00 | 24.2 | 0.360 | 26.1 | 575.00 | 1.2 | 2.23 |
| 160.00 | 320.00 | 25.00 | 0.21 | 74.00 | 2500.00 | 0.330 | 1.70 |

| | | | | | | | |
|---|---|---|---|---|---|---|---|
| 160.00 | 320.00 | 25.00 | 0.21 | 74.00 | 2500.00 | 0.330 | 1.69 |
| 160.00 | 320.00 | 25.00 | 0.21 | 74.00 | 2500.00 | 0.330 | 1.71 |
| 160.00 | 320.00 | 40.00 | 0.24 | 74.00 | 2500.00 | 0.220 | 0.53 |
| 160.00 | 320.00 | 40.00 | 0.24 | 74.00 | 2500.00 | 0.220 | 0.47 |
| 160.00 | 320.00 | 40.00 | 0.24 | 74.00 | 2500.00 | 0.220 | 0.50 |
| 160.00 | 320.00 | 40.00 | 0.24 | 74.00 | 2500.00 | 0.330 | 0.63 |
| 160.00 | 320.00 | 40.00 | 0.24 | 74.00 | 2500.00 | 0.330 | 0.58 |
| 160.00 | 320.00 | 40.00 | 0.24 | 74.00 | 2500.00 | 0.330 | 0.64 |
| 160.00 | 320.00 | 40.00 | 0.24 | 74.00 | 2500.00 | 0.550 | 1.05 |
| 160.00 | 320.00 | 40.00 | 0.24 | 74.00 | 2500.00 | 0.550 | 1.24 |
| 160.00 | 320.00 | 40.00 | 0.24 | 74.00 | 2500.00 | 0.550 | 1.17 |
| 160.00 | 320.00 | 52.00 | 0.25 | 74.00 | 2500.00 | 0.495 | 0.53 |
| 160.00 | 320.00 | 52.00 | 0.25 | 74.00 | 2500.00 | 0.495 | 1.13 |
| 160.00 | 320.00 | 52.00 | 0.25 | 74.00 | 2500.00 | 0.495 | 1.13 |
| 150.00 | 300.00 | 32.54 | 0.248 | 65.00 | 1700.00 | 0.46 | 3.73 |
| 150.00 | 300.00 | 32.54 | 0.248 | 65.00 | 1700.00 | 0.46 | 3.93 |
| 150.00 | 300.00 | 32.54 | 0.248 | 65.00 | 1700.00 | 0.46 | 2.85 |
| 150.00 | 300.00 | 32.54 | 0.248 | 65.00 | 1700.00 | 1.15 | 4.28 |
| 150.00 | 300.00 | 32.54 | 0.248 | 65.00 | 1700.00 | 1.15 | 4.04 |
| 150.00 | 300.00 | 32.54 | 0.248 | 65.00 | 1700.00 | 1.15 | 4.84 |
| 150.00 | 300.00 | 39.00 | 0.23 | 65.00 | 1700.00 | 0.56 | 2.30 |
| 150.00 | 300.00 | 39.00 | 0.23 | 65.00 | 1700.00 | 0.56 | 2.10 |
| 152.00 | 305.00 | 47.8 | 0.222 | 22.00 | 508.2 | 1.25 | 1.35 |
| 152.00 | 305.00 | 47.8 | 0.222 | 22.00 | 508.2 | 1.25 | 1.15 |
| 152.00 | 305.00 | 47.8 | 0.222 | 22.00 | 508.2 | 2.5 | 2.21 |
| 152.00 | 305.00 | 47.8 | 0.222 | 22.00 | 508.2 | 2.5 | 2.21 |
| 152.00 | 305.00 | 47.8 | 0.222 | 22.00 | 508.2 | 3.75 | 2.85 |
| 152.00 | 305.00 | 47.8 | 0.222 | 22.00 | 508.2 | 3.75 | 2.80 |
| 152.00 | 305.00 | 32.2 | 0.22 | 10.50 | 220.00 | 3.00 | 2.04 |
| 152.00 | 305.00 | 32.2 | 0.22 | 10.50 | 220.00 | 3.00 | 1.97 |
| 152.00 | 305.00 | 31.8 | 0.28 | 4.90 | 75.00 | 3.00 | 0.65 |
| 152.00 | 305.00 | 31.8 | 0.28 | 4.90 | 75.00 | 9.00 | 0.95 |
| 152.00 | 305.00 | 32.1 | 0.28 | 4.90 | 75.00 | 1.00 | 0.44 |
| 152.00 | 305.00 | 32.1 | 0.28 | 4.90 | 75.00 | 2.00 | 0.40 |
| 152.00 | 305.00 | 32.1 | 0.28 | 4.90 | 75.00 | 3.00 | 0.50 |
| 152.00 | 305.00 | 32.1 | 0.28 | 4.90 | 75.00 | 6.00 | 0.57 |
| 152.00 | 305.00 | 32.1 | 0.28 | 4.90 | 75.00 | 9.00 | 0.68 |
| 152.00 | 305.00 | 32.1 | 0.28 | 4.90 | 75.00 | 12.00 | 0.82 |
| 152.00 | 305.00 | 32.1 | 0.28 | 4.90 | 75.00 | 15.00 | 0.87 |
| 152.00 | 305.00 | 33.1 | 0.309 | 80.10 | 1826.00 | 0.17 | 1.30 |
| 152.00 | 305.00 | 33.1 | 0.309 | 80.10 | 1826.00 | 0.17 | 1.27 |
| 152.00 | 305.00 | 45.9 | 0.243 | 80.10 | 1826.00 | 0.17 | 0.81 |
| 152.00 | 305.00 | 45.9 | 0.243 | 80.10 | 1826.00 | 0.17 | 1.06 |
| 152.00 | 305.00 | 45.9 | 0.243 | 80.10 | 1826.00 | 0.34 | 1.20 |
| 152.00 | 305.00 | 45.9 | 0.243 | 80.10 | 1826.00 | 0.34 | 1.25 |
| 152.00 | 305.00 | 45.9 | 0.243 | 80.10 | 1826.00 | 0.51 | 1.55 |
| 152.00 | 305.00 | 45.9 | 0.243 | 80.10 | 1826.00 | 0.51 | 1.90 |
| 152.00 | 305.00 | 38.5 | 0.223 | 22.46 | 450.00 | 1.27 | 1.32 |
| 152.00 | 305.00 | 38.5 | 0.223 | 22.46 | 450.00 | 1.27 | 1.46 |

| | | | | | | | |
|---|---|---|---|---|---|---|---|
| 152.00 | 305.00 | 38.5 | 0.223 | 22.46 | 450.00 | 2.54 | 2.46 |
| 152.00 | 305.00 | 38.5 | 0.223 | 22.46 | 450.00 | 2.54 | 2.19 |
| 103.00 | 200.00 | 30.7 | 0.270 | 26.1 | 575.00 | 1.3 | 1.54 |
| 105.00 | 200.00 | 30.7 | 0.270 | 26.1 | 575.00 | 2.6 | 2.75 |
| 103.00 | 200.00 | 46.3 | 0.230 | 26.1 | 575.00 | 1.3 | 0.90 |
| 105.00 | 200.00 | 46.3 | 0.230 | 26.1 | 575.00 | 2.6 | 1.48 |
| 103.00 | 200.00 | 54.5 | 0.240 | 26.1 | 575.00 | 1.3 | 0.32 |
| 105.00 | 200.00 | 54.5 | 0.240 | 26.1 | 575.00 | 2.6 | 0.80 |
| 152.5 | 305.00 | 31.2 | 0.22 | 55.85 | 1800.00 | 0.991 | 3.01 |
| 152.5 | 305.00 | 31.2 | 0.22 | 55.85 | 1800.00 | 0.991 | 3.13 |
| 152.5 | 305.00 | 31.2 | 0.22 | 55.85 | 1800.00 | 1.65 | 5.27 |
| 152.5 | 305.00 | 31.2 | 0.22 | 55.85 | 1800.00 | 1.65 | 6.25 |
| 102.00 | 204.00 | 32.0 | 0.14 | 72.00 | 1520.00 | 0.35 | 1.25 |
| 152.5 | 305.00 | 29.8 | 0.22 | 55.85 | 1800.00 | 0.275 | 1.00 |
| 152.5 | 305.00 | 29.8 | 0.22 | 55.85 | 1800.00 | 0.275 | 1.30 |
| 152.5 | 305.00 | 29.8 | 0.22 | 55.85 | 1800.00 | 0.275 | 1.50 |
| 152.5 | 305.00 | 29.8 | 0.22 | 55.85 | 1800.00 | 0.826 | 2.70 |
| 152.5 | 305.00 | 29.8 | 0.22 | 55.85 | 1800.00 | 0.826 | 1.80 |
| 152.5 | 305.00 | 29.8 | 0.22 | 55.85 | 1800.00 | 0.826 | 3.30 |
| 152.5 | 305.00 | 29.8 | 0.22 | 55.85 | 1800.00 | 1.38 | 3.30 |
| 152.5 | 305.00 | 29.8 | 0.22 | 55.85 | 1800.00 | 1.38 | 3.60 |
| 152.5 | 305.00 | 29.8 | 0.22 | 55.85 | 1800.00 | 1.38 | 3.80 |
| 152.5 | 305.00 | 31.2 | 0.22 | 55.85 | 1800.00 | 0.826 | 3.10 |
| 152.5 | 305.00 | 31.2 | 0.22 | 55.85 | 1800.00 | 0.826 | 3.10 |
| 152.5 | 305.00 | 31.2 | 0.22 | 55.85 | 1800.00 | 1.38 | 4.30 |
| 152.5 | 305.00 | 31.2 | 0.22 | 55.85 | 1800.00 | 1.38 | 5.00 |
| 150.00 | 300.00 | 28.35 | 0.49 | 65.00 | 1700.00 | 0.23 | 1.90 |
| 150.00 | 300.00 | 36.3 | 0.23 | 72.59 | 3240.00 | 0.215 | 2.29 |
| 150.00 | 300.00 | 36.3 | 0.23 | 72.59 | 3240.00 | 0.215 | 1.89 |
| 150.00 | 300.00 | 36.3 | 0.23 | 72.59 | 3240.00 | 0.43 | 3.08 |
| 150.00 | 300.00 | 36.3 | 0.23 | 72.59 | 3240.00 | 0.43 | 3.41 |
| 150.00 | 300.00 | 36.3 | 0.23 | 72.59 | 3240.00 | 0.43 | 2.74 |
| 150.00 | 300.00 | 36.3 | 0.23 | 72.59 | 3240.00 | 0.43 | 2.89 |
| 150.00 | 300.00 | 36.3 | 0.23 | 72.59 | 3240.00 | 0.43 | 3.10 |
| 150.00 | 300.00 | 36.3 | 0.23 | 72.59 | 3240.00 | 0.43 | 2.49 |
| 150.00 | 300.00 | 36.3 | 0.23 | 72.59 | 3240.00 | 0.43 | 2.97 |
| 150.00 | 300.00 | 36.3 | 0.23 | 72.59 | 3240.00 | 0.86 | 3.15 |
| 150.00 | 300.00 | 36.3 | 0.23 | 72.59 | 3240.00 | 0.86 | 4.15 |
| 150.00 | 300.00 | 36.3 | 0.23 | 72.59 | 3240.00 | 0.86 | 4.10 |
| 150.00 | 300.00 | 36.3 | 0.23 | 72.59 | 3240.00 | 1.72 | 5.24 |
| 150.00 | 300.00 | 36.3 | 0.23 | 72.59 | 3240.00 | 1.72 | 5.45 |
| 150.00 | 300.00 | 36.3 | 0.23 | 72.59 | 3240.00 | 1.72 | 4.51 |
| 150.00 | 300.00 | 31.1 | 0.240 | 26.1 | 575.00 | 2.6 | 2.61 |
| 150.00 | 300.00 | 29.6 | 0.240 | 26.1 | 575.00 | 2.6 | 2.72 |
| 150.00 | 300.00 | 31.1 | 0.240 | 26.1 | 575.00 | 2.6 | 2.28 |
| 150.00 | 450.00 | 31.1 | 0.240 | 26.1 | 575.00 | 2.6 | 2.34 |
| 150.00 | 450.00 | 29.6 | 0.240 | 26.1 | 575.00 | 2.6 | 2.32 |
| 150.00 | 450.00 | 31.2 | 0.240 | 26.1 | 575.00 | 2.6 | 2.31 |
| 250.00 | 750.00 | 31.2 | 0.240 | 26.1 | 575.00 | 2.6 | 1.09 |

| | | | | | | | |
|---|---|---|---|---|---|---|---|
| 150.00 | 300.00 | 44.7 | 0.24 | 73.00 | 2300.00 | 0.308 | 0.23 |
| 152.5 | 305.00 | 39.6 | 0.263 | 80.10 | 1826.00 | 0.17 | 0.94 |
| 152.5 | 305.00 | 39.6 | 0.263 | 80.10 | 1826.00 | 0.17 | 0.83 |
| 152.5 | 305.00 | 39.6 | 0.263 | 80.10 | 1826.00 | 0.34 | 2.13 |
| 152.5 | 305.00 | 39.6 | 0.263 | 80.10 | 1826.00 | 0.34 | 1.83 |
| 152.5 | 305.00 | 39.6 | 0.263 | 80.10 | 1826.00 | 0.51 | 2.56 |
| 152.5 | 305.00 | 39.6 | 0.263 | 80.10 | 1826.00 | 0.51 | 1.79 |
| 406.4 | 812.8 | 29.4 | 0.24 | 18.47 | 424.7 | 7.27 | 1.53 |
| 406.4 | 812.8 | 29.4 | 0.24 | 18.47 | 424.7 | 7.27 | 1.45 |
| 406.4 | 812.8 | 29.4 | 0.24 | 18.47 | 424.7 | 7.27 | 1.39 |
| 406.4 | 812.8 | 29.4 | 0.24 | 18.47 | 424.7 | 4.47 | 1.35 |
| 406.4 | 812.8 | 29.4 | 0.24 | 18.47 | 424.7 | 4.47 | 1.00 |
| 406.4 | 812.8 | 29.4 | 0.24 | 18.47 | 424.7 | 4.47 | 1.19 |
| 406.4 | 812.8 | 29.4 | 0.24 | 18.47 | 424.7 | 3.35 | 0.97 |
| 406.4 | 812.8 | 29.4 | 0.24 | 18.47 | 424.7 | 3.35 | 0.90 |
| 406.4 | 812.8 | 29.4 | 0.24 | 18.47 | 424.7 | 3.35 | 0.91 |
| 406.4 | 812.8 | 29.4 | 0.24 | 18.47 | 424.7 | 1.68 | 0.78 |
| 406.4 | 812.8 | 29.4 | 0.24 | 18.47 | 424.7 | 1.68 | 0.70 |
| 406.4 | 812.8 | 29.4 | 0.24 | 18.47 | 424.7 | 1.68 | 0.72 |
| 152.4 | 304.8 | 44.1 | 0.24 | 18.47 | 424.7 | 3.35 | 2.01 |
| 152.4 | 304.8 | 44.1 | 0.24 | 18.47 | 424.7 | 3.35 | 2.01 |
| 152.4 | 304.8 | 44.1 | 0.24 | 18.47 | 424.7 | 3.35 | 2.01 |
| 152.4 | 304.8 | 44.1 | 0.24 | 18.47 | 424.7 | 2.24 | 1.52 |
| 152.4 | 304.8 | 44.1 | 0.24 | 18.47 | 424.7 | 2.24 | 1.49 |
| 152.4 | 304.8 | 44.1 | 0.24 | 18.47 | 424.7 | 2.24 | 1.53 |
| 152.4 | 304.8 | 44.1 | 0.24 | 18.47 | 424.7 | 1.68 | 1.30 |
| 152.4 | 304.8 | 44.1 | 0.24 | 18.47 | 424.7 | 1.68 | 1.36 |
| 152.4 | 304.8 | 44.1 | 0.240 | 18.47 | 424.7 | 1.68 | 1.30 |
| 152.00 | 305.00 | 39.2 | 0.23 | 78.00 | 2400.00 | 0.169 | 2.33 |
| 152.00 | 305.00 | 39.2 | 0.23 | 78.00 | 2400.00 | 0.169 | 2.33 |
| 152.00 | 305.00 | 39.2 | 0.23 | 78.00 | 2400.00 | 0.169 | 2.07 |
| 152.00 | 305.00 | 39.2 | 0.23 | 78.00 | 2400.00 | 0.338 | 3.80 |
| 152.00 | 305.00 | 39.2 | 0.23 | 78.00 | 2400.00 | 0.338 | 3.45 |
| 152.00 | 305.00 | 39.2 | 0.23 | 78.00 | 2400.00 | 0.338 | 3.68 |
| 152.00 | 305.00 | 39.2 | 0.23 | 78.00 | 2400.00 | 0.507 | 4.39 |
| 152.00 | 305.00 | 39.2 | 0.23 | 78.00 | 2400.00 | 0.507 | 4.60 |
| 152.00 | 305.00 | 39.2 | 0.23 | 78.00 | 2400.00 | 0.507 | 4.78 |
| 150.00 | 300.00 | 35.6 | 0.23 | 127.5 | 2640.00 | 2.16 | 9.63 |
| 150.00 | 300.00 | 35.6 | 0.23 | 127.5 | 2640.00 | 2.16 | 6.78 |
| 152.00 | 305.00 | 39.00 | 0.23 | 120.00 | 2900.00 | 0.4 | 2.32 |
| 152.00 | 305.00 | 39.00 | 0.23 | 120.00 | 2900.00 | 0.4 | 2.30 |
| 152.00 | 305.00 | 39.00 | 0.23 | 120.00 | 2900.00 | 0.6 | 3.11 |
| 152.00 | 305.00 | 39.00 | 0.23 | 120.00 | 2900.00 | 0.6 | 2.86 |
| 150.00 | 300.00 | 43.00 | 0.24 | 13.6 | 230.00 | 1.27 | 1.11 |
| 150.00 | 300.00 | 43.00 | 0.24 | 13.6 | 230.00 | 2.56 | 1.47 |
| 150.00 | 300.00 | 43.00 | 0.24 | 13.6 | 230.00 | 3.86 | 1.69 |
| 150.00 | 300.00 | 43.00 | 0.24 | 13.6 | 230.00 | 5.21 | 1.74 |
| 150.00 | 300.00 | 44.7 | 0.24 | 120.00 | 2900.00 | 0.193 | 0.24 |
| 150.00 | 300.00 | 44.7 | 0.24 | 120.00 | 2900.00 | 0.386 | 1.14 |

| | | | | | | | |
|---|---|---|---|---|---|---|---|
| 150.00 | 300.00 | 44.7 | 0.24 | 120.00 | 2900.00 | 0.579 | 1.30 |
| 150.00 | 300.00 | 44.7 | 0.24 | 120.00 | 2900.00 | 0.772 | 1.78 |
| 150.00 | 300.00 | 36.2 | 0.23 | 120.00 | 2900.00 | 0.193 | 0.66 |
| 150.00 | 300.00 | 36.2 | 0.23 | 120.00 | 2900.00 | 0.386 | 1.01 |
| 150.00 | 300.00 | 36.2 | 0.23 | 120.00 | 2900.00 | 0.579 | 1.30 |
| 150.00 | 300.00 | 33.3 | 0.23 | 120.00 | 2900.00 | 0.193 | 0.79 |
| 150.00 | 300.00 | 33.3 | 0.23 | 120.00 | 2900.00 | 0.386 | 1.30 |
| 150.00 | 300.00 | 33.3 | 0.23 | 120.00 | 2900.00 | 0.579 | 1.50 |
| 150.00 | 300.00 | 54.00 | 0.25 | 120.00 | 2900.00 | 0.193 | 0.34 |
| 150.00 | 300.00 | 54.00 | 0.25 | 120.00 | 2900.00 | 0.386 | 0.64 |
| 150.00 | 300.00 | 54.00 | 0.25 | 120.00 | 2900.00 | 0.579 | 0.82 |
| 152.00 | 305.00 | 49.4 | 0.25 | 120.00 | 2900.00 | 0.6 | 3.73 |
| 152.00 | 305.00 | 49.4 | 0.25 | 120.00 | 2900.00 | 0.6 | 3.40 |
| 152.00 | 305.00 | 49.4 | 0.25 | 120.00 | 2900.00 | 0.6 | 3.37 |
| 152.00 | 305.00 | 49.4 | 0.25 | 120.00 | 2900.00 | 0.6 | 3.41 |
| 152.00 | 305.00 | 49.4 | 0.25 | 120.00 | 2900.00 | 0.6 | 3.22 |
| 152.00 | 305.00 | 49.4 | 0.25 | 120.00 | 2900.00 | 0.6 | 3.48 |
| 70.00 | 210.00 | 51.63 | 0.248 | 118.00 | 2060.00 | 0.057 | 0.40 |
| 70.00 | 210.00 | 51.63 | 0.248 | 118.00 | 2060.00 | 0.095 | 0.53 |
| 70.00 | 210.00 | 51.63 | 0.248 | 118.00 | 2060.00 | 0.191 | 0.57 |
| 105.00 | 315.00 | 50.64 | 0.244 | 118.00 | 2060.00 | 0.072 | 0.33 |
| 105.00 | 315.00 | 50.64 | 0.244 | 118.00 | 2060.00 | 0.143 | 0.39 |
| 105.00 | 315.00 | 50.64 | 0.244 | 118.00 | 2060.00 | 0.286 | 0.42 |
| 194.00 | 582.00 | 44.92 | 0.260 | 118.00 | 2060.00 | 0.143 | 0.36 |
| 194.00 | 582.00 | 44.92 | 0.260 | 118.00 | 2060.00 | 0.286 | 0.39 |
| 194.00 | 582.00 | 44.92 | 0.260 | 118.00 | 2060.00 | 0.572 | 0.46 |
| 70.00 | 210.00 | 29.37 | 0.203 | 118.00 | 2060.00 | 0.095 | 0.54 |
| 70.00 | 210.00 | 29.37 | 0.203 | 118.00 | 2060.00 | 0.057 | 0.36 |
| 70.00 | 210.00 | 29.37 | 0.203 | 118.00 | 2060.00 | 0.191 | 0.95 |
| 105.00 | 315.00 | 28.79 | 0.202 | 118.00 | 2060.00 | 0.072 | 0.36 |
| 105.00 | 315.00 | 28.79 | 0.202 | 118.00 | 2060.00 | 0.143 | 0.58 |
| 105.00 | 315.00 | 28.79 | 0.202 | 118.00 | 2060.00 | 0.286 | 1.15 |
| 194.00 | 582.00 | 23.98 | 0.207 | 118.00 | 2060.00 | 0.143 | 0.38 |
| 150.00 | 300.00 | 32.54 | 0.25 | 390.00 | 3000.00 | 0.17 | 0.83 |
| 150.00 | 300.00 | 32.54 | 0.25 | 390.00 | 3000.00 | 0.17 | 0.93 |
| 150.00 | 300.00 | 32.54 | 0.25 | 390.00 | 3000.00 | 0.17 | 0.83 |
| 150.00 | 300.00 | 32.54 | 0.25 | 390.00 | 3000.00 | 0.50 | 1.82 |
| 150.00 | 300.00 | 32.54 | 0.25 | 390.00 | 3000.00 | 0.50 | 1.27 |
| 150.00 | 300.00 | 32.54 | 0.25 | 390.00 | 3000.00 | 0.50 | 1.69 |
| 152.00 | 305.00 | 45.70 | 0.24 | 436.00 | 3314.00 | 0.16 | 1.11 |
| 152.00 | 305.00 | 45.70 | 0.24 | 436.00 | 3314.00 | 0.16 | 1.03 |
| 152.00 | 305.00 | 45.70 | 0.24 | 436.00 | 3314.00 | 0.33 | 1.33 |
| 152.00 | 305.00 | 45.70 | 0.24 | 436.00 | 3314.00 | 0.33 | 1.23 |
| 152.00 | 305.00 | 45.70 | 0.24 | 436.00 | 3314.00 | 0.49 | 1.56 |
| 152.00 | 305.00 | 45.70 | 0.24 | 436.00 | 3314.00 | 0.49 | 1.43 |
| 200.00 | 600.00 | 41.70 | 0.34 | 439.00 | 3972.00 | 0.68 | 1.50 |
| 150.00 | 300.00 | 25.20 | 0.31 | 377.00 | 4410.00 | 0.17 | 1.44 |
| 150.00 | 300.00 | 25.20 | 0.31 | 377.00 | 4410.00 | 0.17 | 1.21 |
| 150.00 | 300.00 | 25.20 | 0.31 | 377.00 | 4410.00 | 0.34 | 1.88 |

| | | | | | | | |
|---|---|---|---|---|---|---|---|
| 150.00 | 300.00 | 25.20 | 0.31 | 377.00 | 4410.00 | 0.34 | 2.10 |
| 150.00 | 300.00 | 25.20 | 0.31 | 377.00 | 4410.00 | 0.51 | 2.45 |
| 150.00 | 300.00 | 25.20 | 0.31 | 377.00 | 4410.00 | 0.51 | 2.43 |
| 150.00 | 300.00 | 47.40 | 0.31 | 377.00 | 4410.00 | 0.17 | 1.09 |
| 150.00 | 300.00 | 47.40 | 0.31 | 377.00 | 4410.00 | 0.17 | 0.87 |
| 150.00 | 300.00 | 47.40 | 0.31 | 377.00 | 4410.00 | 0.34 | 1.40 |
| 150.00 | 300.00 | 47.40 | 0.31 | 377.00 | 4410.00 | 0.34 | 1.35 |
| 150.00 | 300.00 | 47.40 | 0.31 | 377.00 | 4410.00 | 0.51 | 1.59 |
| 150.00 | 300.00 | 47.40 | 0.31 | 377.00 | 4410.00 | 0.51 | 1.69 |
| 150.00 | 300.00 | 51.80 | 0.30 | 377.00 | 4410.00 | 0.17 | 0.75 |
| 150.00 | 300.00 | 51.80 | 0.30 | 377.00 | 4410.00 | 0.17 | 0.66 |
| 150.00 | 300.00 | 51.80 | 0.30 | 377.00 | 4410.00 | 0.34 | 1.05 |
| 150.00 | 300.00 | 51.80 | 0.30 | 377.00 | 4410.00 | 0.34 | 1.00 |
| 150.00 | 300.00 | 51.80 | 0.30 | 377.00 | 4410.00 | 0.51 | 1.29 |
| 150.00 | 300.00 | 51.80 | 0.30 | 377.00 | 4410.00 | 0.51 | 1.20 |
| 150.00 | 300.00 | 51.80 | 0.30 | 377.00 | 4410.00 | 0.85 | 1.59 |
| 150.00 | 300.00 | 51.80 | 0.30 | 377.00 | 4410.00 | 0.85 | 1.61 |
| 150.00 | 300.00 | 34.90 | 0.21 | 640.00 | 2650.00 | 0.24 | 0.40 |
| 150.00 | 300.00 | 44.70 | 0.24 | 640.00 | 2650.00 | 0.38 | 0.53 |
| 100.00 | 200.00 | 30.20 | 0.23 | 637.00 | 2452.00 | 0.14 | 0.57 |
| 100.00 | 200.00 | 30.20 | 0.23 | 637.00 | 2452.00 | 0.28 | 0.88 |
| 100.00 | 200.00 | 30.20 | 0.23 | 637.00 | 2452.00 | 0.42 | 1.30 |
| 150.00 | 300.00 | 23.10 | 0.27 | 540.00 | 1900.00 | 0.29 | 1.27 |
| 150.00 | 300.00 | 23.10 | 0.27 | 540.00 | 1900.00 | 0.29 | 1.20 |

Note: X1 (mm) = column height; X2 (mm) = column diameter; X3 (MPa) = compressive strength of control concrete; X4 (%) = ultimate strain of control concrete; X5 (GPa) = FRP elastic modulus; X6 (MPa) = FRP tensile strength; X7 (mm) = FRP total thickness; Y (%) = ultimate axial strain of FRP-CC columns
